# Supplementary material for: A global assessment of street-network sprawl
Source: PLoS One. 2019 Nov 26;14(11):e0223078. doi: 10.1371/journal.pone.0223078 (PMC6879130; doi:10.1371/journal.pone.0223078)
Supplement: S1 File — (PDF) [file pone.0223078.s001.pdf]

Supporting Information:
A global assessment of street-network sprawl

Christopher Barrington-Leigh[1,◕], Adam Millard-Ball[2,◕],

PLOS One 2019 DOI: 10.1371/journal.pone.0223078

1 McGill University; Institute for Health and Social Policy; and McGill School of Environment; Montreal, Canada
2 University of California Santa Cruz; Environmental Studies Department; Santa Cruz, California

◕Authors are listed alphabetically and contributed equally

Contents

A Measuring street-network sprawl **4**
A.1 Nodal degree . . . 4
A.2 Dendricity . . . 6
A.3 Circuity . . . 6
A.4 Nodal sparsity . . . 7
A.5 Sinuosity . . . 7
A.6 Principal components analysis (PCA) and definition of SNDi . . . 8
A.7 Correlations between metrics . . . 10

B Data sources **10**
B.1 Street-network data . . . 10
B.2 Automobility data . . . 11
B.3 Geographic boundaries . . . 11
B.3.1 National and sub-national administrative regions and world regions . . . 13
B.3.2 Urban boundaries for 200 metropolitan areas . . . 13
B.3.3 Geographic grid . . . 13
B.3.4 Urban region identification . . . 13

C Computational approach **16**
C.1 Street-network data abstraction . . . 16
C.1.1 Splitting roads into edges: . . . 16
C.1.2 Dropping duplicates: . . . 16
C.1.3 Merging compound intersections: . . . 16

C.1.4 Annealing breaks: 17
C.2 Computation of connectivity measures 18
C.2.1 Partitioning 18
C.2.2 Edge properties 18
C.2.3 Node properties 19

D Street-network paradigms **20**

E Sample grid cells **21**
E.1 High connectivity (low SNDi) 21
E.2 Low connectivity (high SNDi) 21

F Distributions of SNDi **26**

G Empirical street-network types (k-means cluster analysis) **28**

H Automobility regressions **34**

I Sensitivity analysis **34**
I.1 Incompleteness tests 34
I.2 PCA weighting 35
I.3 Buffer radius used to separate nodes/junctions 36
I.4 Exclusion of walking and cycling paths 36

J Data release **38**

K Open-source code **38**

L Citation and contact **38**

References **38**

M Results **41**
M.1 Countries ranked by urban road network sprawl (SNDi) of total road stock 41
M.2 Cities ranked by urban road network sprawl (SNDi) of total road stock 47
M.3 Plots of cluster examples 52
M.4 World maps 63

List of Figures

A	Schematic of selected connectivity measures	4
B	Principal components' explained variance	9
C	Correlations among country-level sprawl metrics and other national variables	12
D	Imputation of urban area boundary for Monterrey, Mexico	15
E	Merging and annealing of nodes	17
F	Distribution of SNDi within the 49 largest countries	27
G	Radar plot of -means cluster analysis	29
H	Distribution of empirical street-network types within 100 largest countries	30
I	Distribution of empirical street-network types within large cities (i)	31
J	Distribution of empirical street-network types within large cities (ii)	32
K	Distribution of vehicle ownership and walking across empirical street-network types, United States.	33
L	Trends in SNDi as a function of completeness	35
M	Robustness of to alternate assumptions.	36
N	Robustness of principal component coefficients to alternate assumptions	37

List of Tables

A	Overview of core and aggregate connectivity measures	5
B	Principal component loadings	9
C	Connectivity metrics for street-network paradigms	20
D	Sample grid cells	23
E	Regressions of vehicle ownership and walking	34
F	Countries ranked by street stock	42
G	Cities ranked by street stock	48

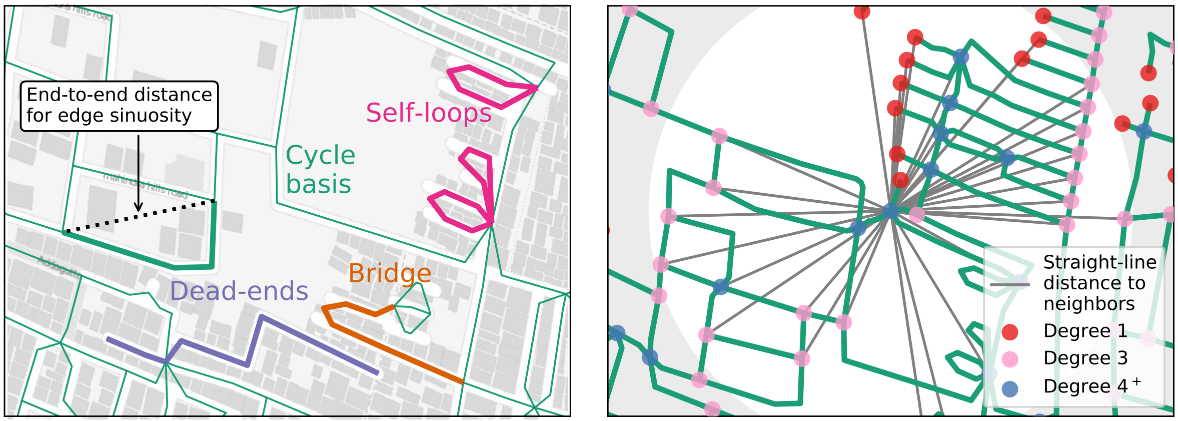

(a) (b)

Figure A: **Schematic of selected connectivity measures**. (a) Edge-related measures include sinuosity, which is the ratio of end-to-end length to path length, and dendricity metrics, which incorporate a classification of all edges into one of four types based on their network function. Solid lines indicate streets. (b) Node-related measures are based on the nodal degree, indicated by colored circles, and circuity, which relates to the shortest path lengths and the direct-path distances between one node and all its neighbors within some radius. Green lines indicate streets, while gray lines indicate straight-line distances to nearby nodes. (This figure also appears in the main text, but is repeated here for clarity of exposition.)

A Measuring street-network sprawl

We measure street-network sprawl through numerous metrics that are derived from graph theoretic and spatial principles. Selected measures are illustrated schematically in Figure A and categorized in Table A. The following subsections discuss each group of metrics in turn, along with the construction of our scalar summary measure, SNDi. Detailed definitions of each metric are provided in the text and Table A.

A.1 Nodal degree

Nodal degree measures: (i) negative nodal degree, (ii) fraction nodes of degree 1, (iii) fraction nodes of degree 1 or 3.

Each node takes a value of 1, 3 or 4+, depending on the number of incoming edges. A cul-de-sac has nodal degree of one, while nodes in a grid are of degree four. We top-code nodal degree as four, so that any node with degree > 4 is assigned a value of four when computing means and other summary statistics.

In the analysis, we normally align our nodal degree measures so that a higher value indicates greater street-network sprawl. Therefore, we use negative nodal degree in place of nodal degree, and the fraction of nodes with degree 1 or degree 3 rather than the fraction with degree-4+.

	Concept	Core metric	Aggregated measure(s)	
Edges	Edge classification (dendricity)	dead-end	Fractions of edges; fraction of length	graph-theoretic
		self-loop		
		other bridge		
		part of a cycle		
	Sinuosity	path length	Gross sinuosity, i.e. summed path length / summed end-to-end length	geographic
		end-to-end length		
Nodes	Nodal degree	dead-end	Mean degree; fraction of dead-ends and fraction of degree<4	graph-theoretic
		degree 3		
		degree>3		
	Node-node distance ratios (circuity)	$r \leq 500$ m	Summed path length over summed end-to-end length for all proximate node pairs within radius r	geographic
		500 m $< r \leq$ 1000 m		
		1000 m $< r \leq$ 1500 m		
		1500 m $< r \leq$ 2000 m		
		2000 m $< r \leq$ 2500 m		
		2500 m $< r \leq$ 3000 m		
	Nodal density	Nodes within 500 m	Node-weighted mean area density	
		Nodes within 1000 m		
		Nodes within 1500 m		
		Nodes within 2000 m		
		Nodes within 2500 m		

Table A: **Overview of core and aggregate connectivity measures.** Our database consists of computed values of the core metrics for each edge and node. When expressing aggregate metrics for administrative or city regions, we use the approach in the "Aggregation measure(s)" column. For our preferred results, we include only the edges and nodes we deem to be urban and that are open to motor vehicles, when aggregating. In order to align our metrics with increasing sprawl, we use sparsity (inverse density) in place of density, and negative degree in place of mean degree.

A.2 Dendricity

Our dendricity measures, derived from graph theory, quantify the extent to which streets form part of a connected network (i.e. a connected graph), versus a tree-like or dendritic network where there is only one possible route to reach other nodes. Each **edge** (i.e., a link or street segment between two nodes) is classified as a bridge, dead-end, self-loop or a member of the cycle basis:

- A **bridge** (also: isthmus, cut-edge, or cut arc) is an edge whose deletion increases the number of components, i.e. completely disconnected subgraphs. (Equivalently, an edge is a bridge if and only if it is not contained in any **cycle**, and is not a dead-end.) Informally, a bridge can be understood as the only route into a given neighborhood, such as the entrance to a gated community or the causeway from the Italian mainland to Venice. Contrary to formal network terminology, our measure of bridges excludes dead-ends.

- A **dead-end** is an edge where one of the nodes has degree one. Formally, dead-ends are also bridges, but we separate out the two categories because they serve different functions on a transportation network.

- A **self-loop** is an edge that joins a node to itself. For example, the "lollipops on a stick" style of urban development [Southworth and Ben-Joseph, 2003] involves self-loops.

- An edge is in the **cycle basis** if it forms part of any *cycle*, i.e. a sequence of edges that can be followed to return to the starting point without visiting any other node more than once. Any edge that is not a bridge, dead-end or self-loop is in the cycle basis.

Our graph-theoretic measures consist of the fractions of edges that are (i) bridges, and (ii) bridges, dead-ends and self-loops (i.e., not in the cycle basis), which Parthasarathi et al. [2015] call "treeness." We compute each as a fraction of the number of edges and as a fraction of edge length. See Marshall et al. [2018] for a detailed discussion of graph theoretic approaches to street-network modeling.

A.3 Circuity

The circuity of a path is the ratio of the shortest network distance to the straight-line distance between start and end points [e.g. Giacomin and Levinson, 2015, Boeing, 2018]. A more circuitous network will involve longer travel distances, compared to straight-line distances. We use the log of circuity in order to generate a metric with a more Gaussian distribution.

It is unclear *a priori* which pairs of nodes to consider when calculating circuity. We do not have activity data in order to weight nodes by their importance, for example, by giving a greater weight to schools, retail and other highly frequented locations. We therefore calculate circuity between an origin node and all destinations within different distance bands of the origin: 0–500m, 500–1000m, 1000–1500m, 1500–2000m, 2000–2500m, and 2500–3000m. This gives us six different measures of circuity, each defined by the distance band, an approach which is likely to mitigate some of the problems of scale-dependency identified by Boeing [2018].

Formally, we define the log circuity LC for a *given path* from node A to node B as

$$\begin{aligned} LC_{AB} &= \log\left(\frac{S_{AB}}{R_{AB}}\right) \\ &= \log S_{AB} - \log R_{AB} \end{aligned}$$

where S_{AB} is the network distance and R_{AB} is the straight-line distance.

Similarly, we define the log circuity for *an origin node* and distance band $[r_1, r_2]$ as:

$$LC_{\text{node},r_1,r_2} = \log\left(\sum_{\substack{\text{node neighbours}\\ r_1<R_{AB}\leq r_2}} S_{AB}\right) - \log\left(\sum_{\substack{\text{node neighbours}\\ r_1<R_{AB}\leq r_2}} R_{AB}\right)$$

and the log circuity for a *region* and distance band $[r_1, r_2]$ as:

$$LC_{r_1,r_2} = \log\left(\sum_{\substack{\text{node pairs}\\ r_1<R_{AB}\leq r_2}} S_{AB}\right) - \log\left(\sum_{\substack{\text{node pairs}\\ r_1<R_{AB}\leq r_2}} R_{AB}\right)$$

A.4 Nodal sparsity

We use nodal sparsity rather than its inverse, nodal density, in order to align our measures of street-network sprawl so that a higher value is associated with more sprawl. We calculate nodal sparsity as a weighted function of individual nodes within a given distance of each node using six distance radii: 500m, 1000, 1500, 2000m, 2500m, and 3000m. This measure is nearly log-normally distributed, so we use its log form. Log nodal sparsity $\langle LNS \rangle_{j,r}$ for geographic unit j and distance radius r is defined as:

$$LNS_{j,r} = \log\left(\frac{N_j \pi r^2}{\sum_{i=i}^{N_j} d_{i,r}}\right)$$

Where $d_{i,r}$ gives the number of destination nodes within distance r of each origin node $i = 1...N_j$ within geographic unit j.

The key advantage of our approach, in contrast to the conventional way of calculating nodal sparsity as $\pi r^2/N_j$, is to reduce the sensitivity of nodal density to the definition of the geographic units, such as the inclusion of a large natural area, lake or other undeveloped land. For an analogous discussion in the context of residential density, see Eidlin [2010]. Another advantage is that we can report LNS for only the nodes classified as urban (see Section §B.3.4) without constructing explicit boundaries for our urban classification.

A.5 Sinuosity

The sinuosity, or curviness, of an edge is the ratio of edge length to the distance between the start and end nodes. A straight edge will have a sinuosity of 1, while a self-loop has infinite sinuosity. When aggregating, we define regional sinuosity as the ratio of the sum of all edge lengths to the sum of straight-line distances between start and end nodes. Unlike our somewhat analogous measure of circuity, sinuosity is a property of individual edges, not the longer multi-edge routes in a network.

A.6 Principal components analysis (PCA) and definition of SNDi

In order to reduce the dimensionality of our street-network sprawl measures, we estimate principal components from thirteen nodal degree, circuity, edge classification, and sinuosity metrics. We estimate principal components using our road network sprawl measures calculated for the highest-resolution geographic regions available from the GADM dataset, which spans the planet and is discussed in Section §B.3.1. We then apply the transformation from this estimate to calculate the first two components using aggregate metrics at all other geographic scales. See I.2 for more on the robustness of the estimate. Table B shows the PCA estimates. The coefficients (loadings) shown are multiplied by normalized versions of the original variables to obtain the PCA vectors. If μ_i and σ_i are the mean and standard deviation of original sprawl metric m_i in the original (high-resolution) data used to calculate the PCA coefficients, then these coefficients c_{ij} are applied to the metrics m'_i in other geographic scales as:

$$\mathrm{PCA}_j = \sum_i c_{ij} \frac{m'_i - \mu_i}{\sigma_i}$$

PCA_1 has the feature that its coefficients are all positive and are relatively evenly loaded across metrics, indicating that our metrics all contribute in the same direction (see Section §A.7 for pairwise correlation coefficients) to our preferred measure of street-network sprawl, and that our varied metrics do capture independent variation in street-network connectivity. Figure B shows the eigenvalues and fraction of explained variance for all PCA components. We consider only the first two components, PCA_1 and PCA_2, which lie well above the canonical heuristic cutoff eigenvalue of 1.

We focus on PCA_1 in our primary analysis, as it captures the bulk of the variation, is well spread across our metrics, and points in the direction of increasing sprawl for each of them. PCA_2, in contrast, emphasizes differences between our network theoretic measures and our spatial ones, rather than pointing unambiguously in the direction of increasing sprawl. PCA_2 loads much more positively on the measures of circuity, with the coefficients for PCA_2 about twice those for PCA_1, and negatively on all other measures. Thus, high PCA_2 can be interpreted as a place where the circuity is much higher than would be expected from nodal degree and other graph-theoretic measures of connectivity. We inspected a range of grid cells that score highly on PCA_2, and most were characterized by neighborhoods with few culs-de-sac but separated from each other by bodies of water, steep ravines, agricultural land, or simply through design. Kuwait and Qatar are the two countries with the highest level of PCA_2 (see, for example, 29°17'00.0 N+47°49'30.0 E).

While PCA_2 may thus be useful for discriminating between different subsets of street-network styles which achieve a given level of PCA_1, it is the PCA_1 measure which best captures the degree of "sprawl" (disconnectedness) from each of our measures. In the remainder of this SI and in the main paper, we refer to PCA_1 as the Street-Network Disconnectedness Index (SNDi). However, in order to make most values positive for convenience, we add a small value to PCA_1: we therefore define $\mathrm{SNDi} = \mathrm{PCA}_1 + 3.0$.

	PCA_1	PCA_2	PCA_3
Nodal degree (negative)	.35	−.12	−.44
Frc deadends	.35	−.12	−.45
Log Circuity (0–0.5km)	.27	.23	−.12
Log Circuity (0.5–1km)	.30	.29	−.041
Log Circuity (1–1.5km)	.29	.33	.030
Log Circuity (1.5–2km)	.25	.37	.11
Log Circuity (2–2.5km)	.19	.38	.21
Log Circuity (2.5–3km)	.11	.27	.32
Frc bridges (length)	.22	−.34	.48
Frc non-cycle (length)	.32	−.31	.17
Frc non-cycle (N edges)	.38	−.26	−.11
Frc bridges (N edges)	.28	−.30	.33
Log(sinuosity)	.16	.033	.21
Variance explained	37%	22%	9%
Eigenvalue	4.9	2.9	1.2

Table B: **Principal component loadings**

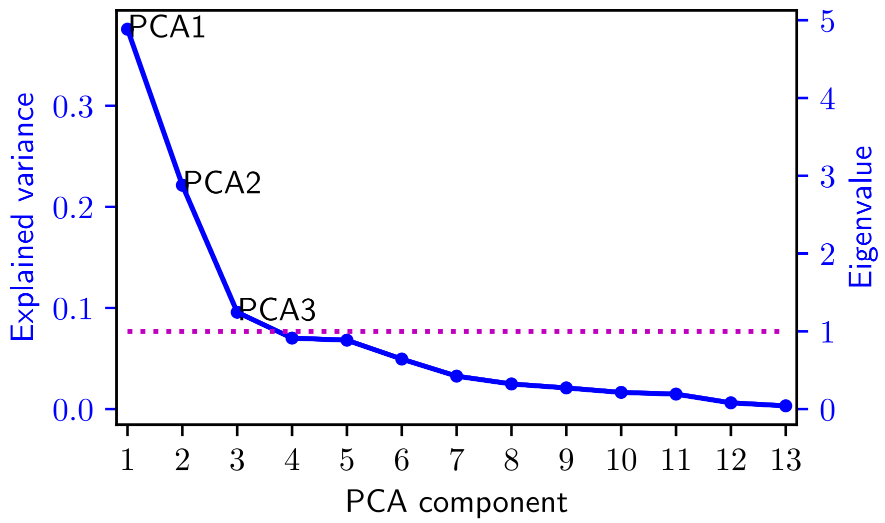

Figure B: **Principal components' explained variance**

A.7 Correlations between metrics

Figure C shows distributions of and pairwise coefficients between our main metrics aggregated to the country level.

B Data sources

In addition to raw street data from OpenStreetMap used to derive our street network, we make use of several open-access geographic boundary data sets, as well as some automobility data in our analysis. These are described below.

B.1 Street-network data

OpenStreetMap (OSM) is a free, community-driven, open-data, volunteer-contributed platform and repository of geographic information covering the whole world. OSM launched in 2004 with a focus on streets, but has subsequently expanded to map buildings, land uses, points of interest and other geographic features [Jokar Arsanjani et al., 2015]. As of November 2018, ∼5.0 million contributors had created a database with ∼537 million roads, coastlines, administrative boundaries and other linear features known as "ways" [OpenStreetMap, 2018].

We make use solely of the road data. There is no universal definition of a "road", and OSM *ways* have numerous tags to help to identify their nature. When computing dendricity, circuity and other measures that rely on graph-theoretic properties, we include in our primary analysis ways with any kind of "highway" tag, except those tagged "track" which primarily indicate paths for agricultural use. However, when aggregating our results, we only include highways sub-categorized as "motorway", "motorway_link", "trunk", "trunk_link", "primary", "primary_link", "secondary", "secondary_link", "tertiary", "tertiary_link", "residential", "road", "unclassified", and "living_street", and exclude those sub-categorized as "service", "pedestrian", "path", "cycleway", "footway", and "steps".

In making this distinction, our aim is to reflect the full range of ways that enhance connectivity for cyclists and pedestrians when measuring the properties of the network. For example, an edge may be a dead-end for motor vehicles, but allow pedestrians to pass freely at either end. Thus we *include* cycleways, footpaths, service roads, and similar ways when c*omputing graph properties* and our measures of dendricity, nodal degree and circuity, but *exclude* them when computing the aggregate measures. Intuitively, a pedestrian or cycle path improves our measure of connectivity indirectly, through its effect on nearby streets.

This procedure helps to avoid several potential biases. For example, sidewalks are sometimes represented in OSM as a separate way that parallels the street; in such a situation, that street would be double counted. The "service" tag, meanwhile, is often used to identify private driveways, a practice that would inflate the fraction of dead ends. "Service" ways also represent other access roads that do not form part of the public street network, and aisles in parking lots, the internal connectivity of which has little relevance to urban form or travel behavior.

Analogously, we exclude nodes where all of the connected edges are excluded from the aggregation. For example, we exclude a node that represents the intersection of two pedestrian paths, but include one that would be a dead-end for cars but a through node for bicycles. We also exclude nodes that would be degree-2 but for the existence of a service road, or a pedestrian path or other edge closed to cars; a typical example here is the intersection of a private driveway with the public street.

For computational reasons, we also drop a small number of ways that cross the -180/+180 line of longitude.

OSM makes its entire global dataset available for download. Our analysis is based on `planet-latest.osm.pbf` [42GB], downloaded from `https://planet.osm.org/` on 8 August 2018.

No map is perfectly up to date nor complete, and in many locations around the world there may be no more authoritative map than OSM. In earlier extensive work, we estimated the completeness of road data in OSM, and found that by mid-2017, the data are nearly 90% complete, globally, with the remaining gaps largely confined to certain areas, in particular China, where there are legal restrictions on mapping, and parts of Africa [updated analysis based on Barrington-Leigh and Millard-Ball, 2017]. As a result, the measures we report in the present work are largely exact measures, rather than estimates, as they include essentially every intersection and every road segment in each unit of geographic aggregation. These estimates, however, do not account for pedestrian paths and similar features, which are less complete in many countries. Therefore, in Section §I.4 we discuss a robustness check in which we limit the analysis to the subset of ways that are open to motor vehicles.

B.2 Automobility data

Our data on vehicle ownership and commute mode share come from official government sources as follows:

- **United States:** American Community Survey data are used for vehicle ownership and commute mode share at the census block group level. We use the 5-year estimates for 2012-16.
- **Japan:** The Ministry of Land, Infrastructure, Transport and Tourism provides vehicle ownership data at the municipality level for purchase at an approximate cost of USD500. No commute mode share are available.
- **New Zealand:** Data on vehicle ownership and commute mode share are from the 2013 census. We use census-defined area units rather than the smallest level of geographic aggregation (meshblocks), to avoid dropping meshblocks where data are not reported in order to preserve individual privacy.
- **France:** Data on vehicle ownership are from the 2015 census, and are provided by L'Institut national de la statistique et des études économiques. We use the data at the smallest level of geographic aggregation: Ilots Regroupés pour l'Information Statistique (IRIS) where available, and municipality ("commune") for smaller municipalities which are not sub-divided by IRIS. Our 49,693 units have a mean size of ∼584 households, with the largest consisting of 5,726 households. Geographic boundary files are from the Institut national de l'information géographique et forestière (IGN).

In all cases, vehicle ownership data are standardized to vehicles per household.

B.3 Geographic boundaries

In addition to the OpenStreetMap street data, we use three different sets of data for geographic boundaries to define regions of interest.

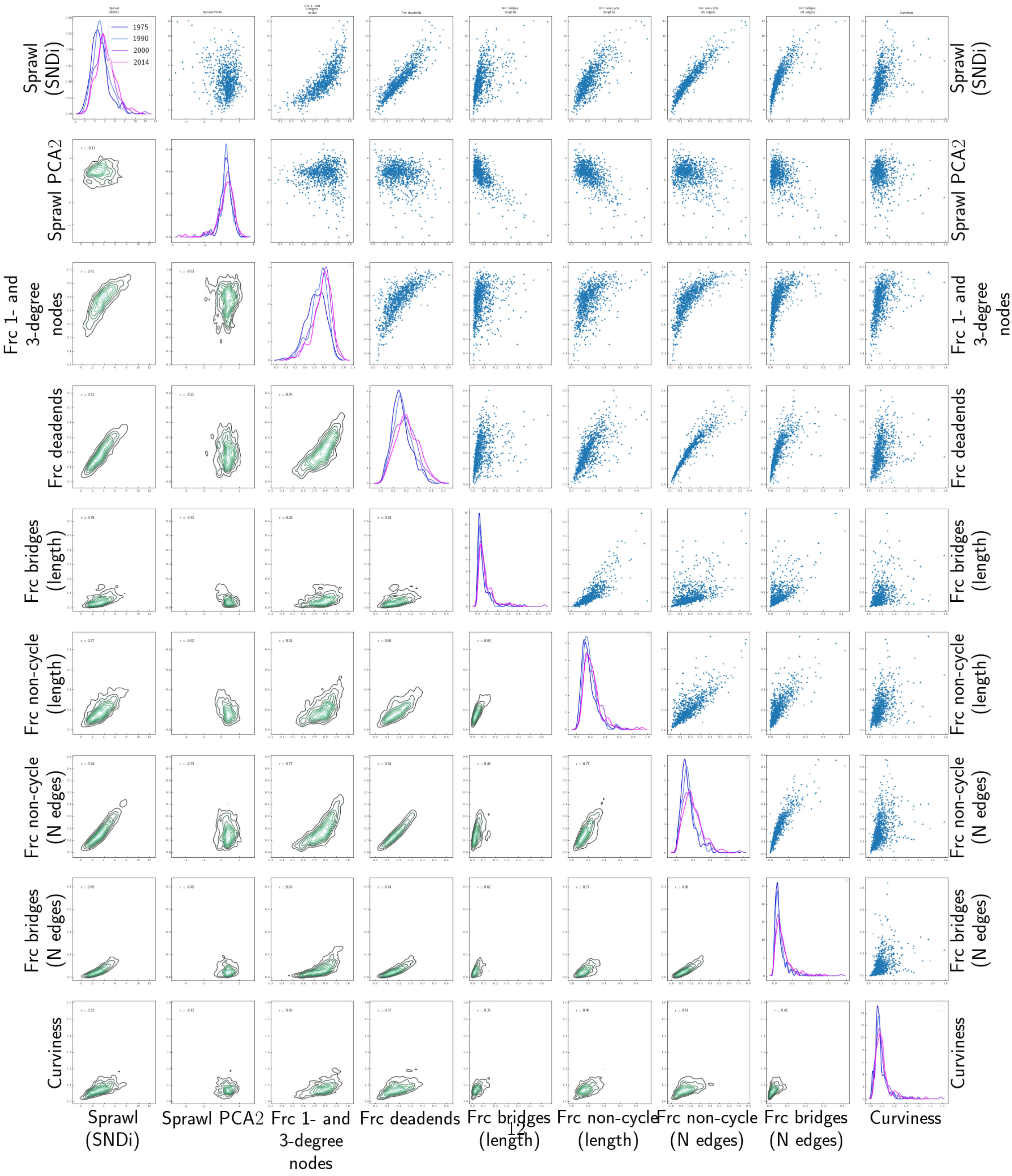

Figure C: **Correlations among country-level sprawl metrics and other national variables**. Frames along the diagonal show distributions of each variable by time period (see top left frame). Frames above/right of the diagonal show bivariate scatter plots of values in each country in each of four time periods. Frames below/left of the diagonal show contour/density plots of this same bivariate distribution. Zoom in on a full resolution electronic version of this figure to see details.

B.3.1 National and sub-national administrative regions and world regions

Administrative units: We use the Global Administrative Areas (GADM) database, v. 3.6 (gadm.org). This provides geographic boundaries for all countries in the world, and up to five levels of sub-national geography such as states, provinces, counties, townships, parishes and prefectures.

World regions: Based on the GADM geographic boundaries for countries, we also aggregate up to geographic, income-based and other country groupings defined by the World Bank.

B.3.2 Urban boundaries for 200 metropolitan areas

We use urban boundary data from the Atlas of Urban Expansion [Angel et al., 2012, 2016], an open-data online database built by the Lincoln Institute of Land Policy, UN-Habitat, and New York University. The Atlas includes a sample of 200 of the world's metropolitan areas that had 100,000 people or more in 2010.

B.3.3 Geographic grid

Our lowest level of geographic aggregation is to a 30-arc second grid, which yields $\sim$1km^2 grid cells at the equator, and smaller cells at high latitudes. We choose the 30-arc second grid in order to align our analysis with the population density data from Landscan, which is provided at the same resolution. See `http://web.ornl.gov/sci/landscan/datasets/LS2012.ris` for restrictions and legal notifications regarding the Landscan data.

B.3.4 Urban region identification

While we compute our measures of street-network sprawl for every node and edge in the world, our main aggregations and analyses are restricted to urbanized areas. There is, however, no standard definition of "urban," and the physical, social and economic characteristics that distinguish the urban from the rural are context dependent. United Nations urban population data are based on national sources, and the criteria for designating urban settlements — not to mention their spatial extent — vary considerably between countries [Cohen, 2006]. While global estimates of population density by grid cell are available (e.g. Laboratory [2013], Zhou et al. [2015]; see Potere and Schneider [2007] for a review), the threshold density that can be considered "urban" varies by country. For example, some isolated villages in developing countries have a higher population density than suburban housing subdivisions in the United States. In Canada, the United States and India, for example, places defined as urban must have a density of at least 400 persons km^{-2}, while the density threshold is 150 persons km^{-2} in Malta, 500 in the Philippines and 1500 in China [United Nations, 2014].

We therefore adopt a multi-stage approach to classify each edge and node as "urban" or "non-urban," using independent data sources and explicitly allowing the urban density threshold to vary by country:

1. We assign a population density to each node and edge, using the Landscan grid [Laboratory, 2013]. Where an edge intersects more than one Landscan grid cell, we take the mean density.
2. We assign a "fraction developed" value to each node and edge, using the GHSL grid of human settlements [Pesaresi et al., 2013]. The "fraction developed" is the fraction of pixels in the GHSL analysis that are built up.

3. We use the Atlas of Urban Expansion (Section §B.3.2) to relate the global Landscan and GHSL datasets to an independent, carefully calibrated urban region boundary. We set the urban density threshold for each country as the 2nd percentile density of nodes that lie within Atlas-defined cities in that country. (Using the 2nd percentile helps to exclude parks and other undeveloped spaces.) We set the "fraction developed" threshold in a similar way using the 2nd percentile, but on a global rather than a country-specific basis, since there is less cross-national variation on this measure.

4. For countries with no Atlas-defined city, we impute the density threshold as the mean of the three most-similar countries, based on geographic proximity, income and urbanization. We define "most similar" as the shortest Mahalanobis distance in a four-dimensional space defined by latitude, longitude, per capita GDP at purchasing power parity, and fraction of population that is urbanized using United Nations measures. For countries with missing data on GDP or urbanized population, we impute using the global mean. 172 of 255 GADM-defined countries use imputed data. However, most of these are principalities, colonies, small island states or non-UN recognized countries; the first five in alphabetical order are Aruba, Anguilla, Åland, Albania and Andorra. In population terms, imputed countries account for 8.3% of the global population (using LandScan population data), and the largest is Peru.

5. For each node and edge, we classify it as urban if its density is weakly greater than (i.e., at least as great as) its country-specific density threshold, *or* if its fraction developed is greater than the global threshold. Using a boolean "OR" criterion means that we are more inclusive in our definition of urban, but we are more robust to errors in either the Landscan or GHSL datasets. Figure D shows an example of how these criteria apply to the city of Monterrey.

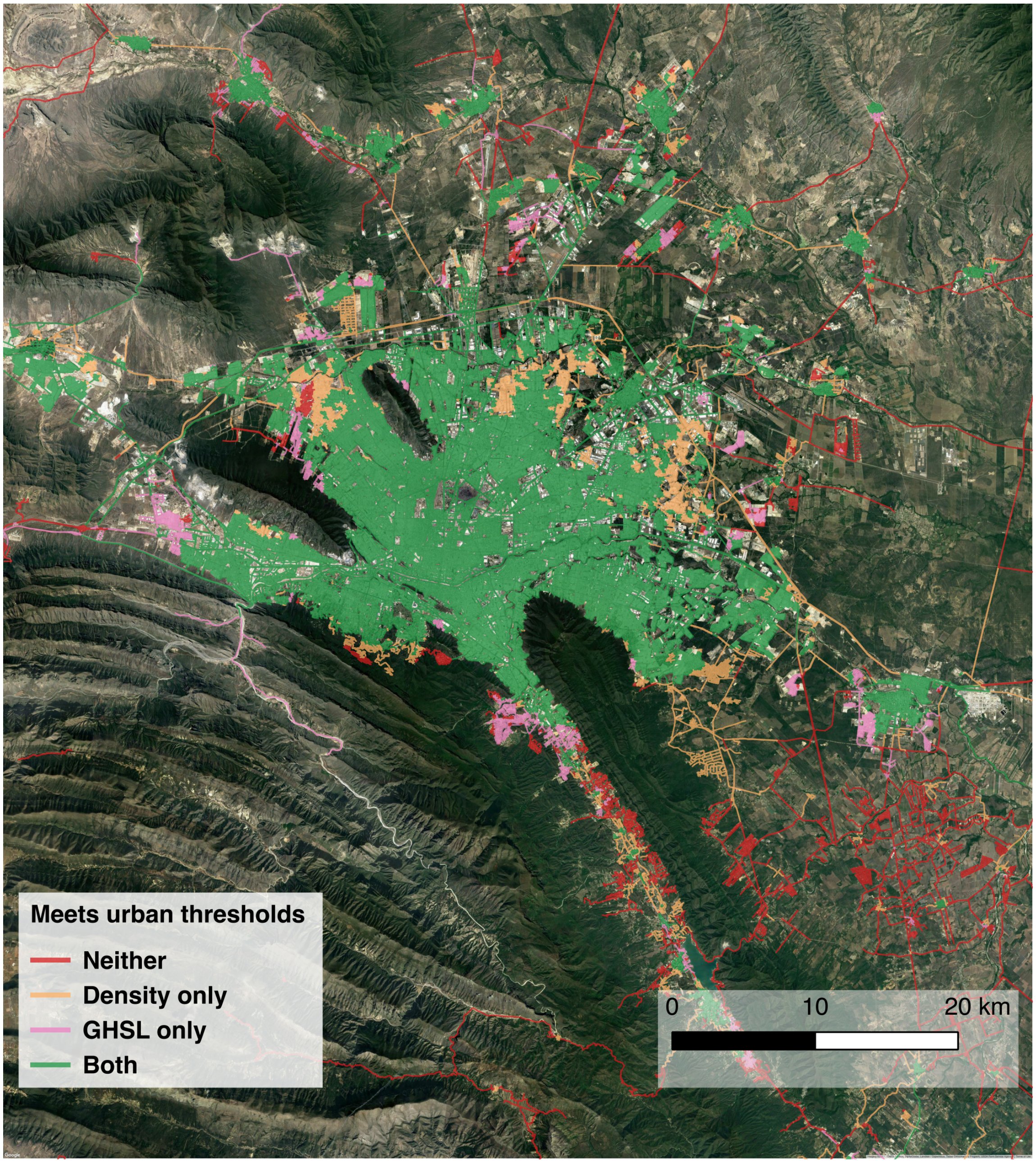

Figure D: **Imputation of urban area boundary for Monterrey, Mexico.** The red-colored edges are below the 2nd percentile density for Mexico (79.9 persons km^{-2}), and also below the 2nd percentile fraction developed in the GHSL dataset (0.094). All other edges are classified as urban. Base imagery source: Google/OpenLayers.

C Computational approach

Our processing was carried out using the Python programming language and a PostgreSQL database augmented with geographic and geometric data types and functions (PostGIS). We made use of moderate parallelization (56 processors) for many tasks, and of the ∼0.7 TB RAM available on our dedicated computation server. Since development is complete, using the extensible resources of a cloud server would likely be the best way to reproduce our work. Except for the *osm2po* package (which is "freeware") all software, including operating systems, used for our calculations is open source. See Section §K for more information on reproducing our work.

C.1 Street-network data abstraction

The raw data requires several pre-processing steps before we are able to calculate our connectivity metrics. Some of these pre-processing steps are designed to deal with the inconsistencies and idiosyncrasies of the OSM database, while others are required to convert the cartographically oriented OSM data into a form amenable to network analysis.

C.1.1 Splitting roads into edges:

We load the raw OSM data into a PostGIS spatial database using the *osm2po* software package (osm2po.de). The *osm2po* stage splits each street into its constituent edges, i.e. into individual street segments between two nodes. The *osm2po* stage also filters out linear features that we do not consider streets. See Section §B.1 for which features we include.

C.1.2 Dropping duplicates:

Possibly our slowest procedure, because we cannot easily parallelize it, is to eliminate duplicate geometries. Approximately 82,000 edges are duplicated in OSM. In some cases, the duplicates reflect mistakes in the OSM database, while in others they result from the import process that converts polygons representing a square, plaza or similar feature into linear streets. We also delete isolated self-loops that have no connections to the larger street network.

C.1.3 Merging compound intersections:

One challenge with the OSM data is that they represent complex intersections with a large number of nodes. For example, a staggered (offset) four-degree intersection would be represented as two three-degree nodes, while dual carriageways and freeway junctions are comprised of many nodes and internal edges. We therefore collapse the OSM nodes into node-clusters (simply referred to as "nodes" in this paper). Nodes within 20m of each other are merged, and the edges are redirected to the centroid of the node-cluster (Figure E). This step is accomplished through our custom algorithms applied to the PostGIS database. A robustness check shows that almost identical results are obtained if we merge nodes within 14m of each other. These distance calculations are performed using geodesic distances, in order to minimize the distortions from using a projected coordinate system.

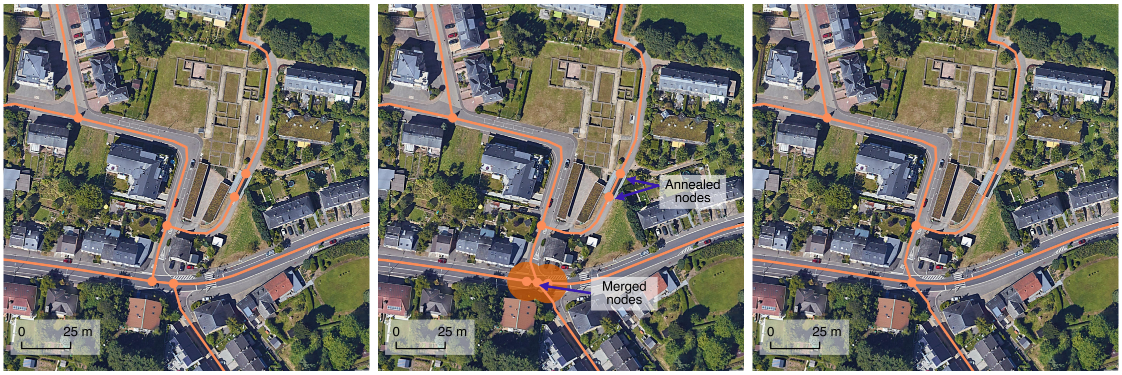

Figure E: **Merging and annealing of nodes.** The left panel shows the raw OSM street network, with two 2-degree nodes and an offset intersection comprised of two 3-degree nodes. (The street between the 2-degree nodes is a bridge that is represented separately in OSM.) The center panel shows the 10m radius around the nodes of the offset intersection, within which the nodes are merged, and the nodes to be annealed. The right panel shows the final processed street network that we use in the analysis. The example is from Helmsange, Luxembourg.

C.1.4 Annealing breaks:

The final pre-processing step is to anneal degree-2 nodes, which by definition do not represent intersections. Their existence is an artefact of the *osm2po* import process or simply reflects the way data have been entered into OSM. For each 2-degree node, we merge the two edges that meet at that node, and delete the node from the database (Figure E).

Our algorithm for accomplishing this is somewhat involved, because (1) in many cases, degree-2 nodes are not isolated, but exist in chains; (2) such chains have the possibility of being loops; and (3) edges are coded with a "start" and an "end" node, so that for any given degree-2 nodes, there are four possible relationships with the adjoining edges. Because of (1) and (2), one cannot safely process a long list of apparent degree-2 nodes with a single pass. We proceed in the following steps:

1. Delete all isolated loops constituted of exactly two parallel (in the graph theoretic sense) edges.
2. Select all edges with degree-2 nodes at *both* ends ("double-degree-2-edges"), build a network data structure from them, and find all connected components (using *networkx*, a network theory package implemented in Python). This gives all the chains consisting solely of degree-2 nodes.
3. These chains are annealed (nodes removed; edges joined) and re-inserted into the database. This replaces each original multi-edge double-degree-2-edge chain with a single double-degree-2-edge.
4. Repeatedly edit the edges/nodes database tables, annealing edges which have only one degree-2 node. This should properly reduce loops to simple self loop edges. When one edge of a pair has two degree-2 nodes, keep its edge_id and drop the other's.

5. Drop isolated self-loops, that is, edges which have a single degree-2 node that connects the start and end points, and are not connected to any other edges.

6. Drop parallel edges that have the same start and end node, and are not open to motor vehicles. Most of these parallel edges consist of sidewalks and walking paths that are represented separately from the street in the OSM database.

C.2 Computation of connectivity measures

Our basic computations are carried out on a PostGIS database with a table of all edges and a table of all nodes. For some network computations, we load the road network, in sections, into a Python data structure rather than working on it directly within the PostGIS database. For this purpose, and for convenience in parallelization, we create a lookup between street features and GADM regions (see Section §B.3.1).

We make use of the *networkx* Python package for generic graph-theoretic operations. We do not use *pgRouting*, which plugs into PostGIS, nor the *osmnx* package [Boeing, 2017] in Python, which has considerable overlap in intent with our application.

For computing distances in the steps below, we use precise geodesic calculations using the PostGIS geography data type. For efficient selection of nodes and edges within buffer regions (see Section §C.1.3), we use a Robinson projection (`http://spatialreference.org/ref/esri/54030`), which provides a compromise between shape and distance distortions.

C.2.1 Partitioning

In our favored procedure, we parallelize the computation of edge and node connectivity measures across overlapping regions. We explored the scaling properties of different aspects of our analysis, some of which scale concavely and some convexly with increasing network size. This analysis suggests a roughly optimal chunk size for applying our connectivity algorithms. Using GADM region boundaries, the network in each country is recursively partitioned into GADM regions containing a sufficiently small number of nodes ($\sim 2 \times 10^4$). For computation of the node and edge properties of each of these GADM "core" regions, all nodes and edges within 10 km of the core region are also included. These buffer regions can include streets in neighboring countries, so that neither our parallelization nor our chunk partitioning should introduce errors in our characterization of nodes or edges.

C.2.2 Edge properties

We build each analysis "chunk" of the network, extracted from our PostGIS database, into a *networkx* graph. We then use the *networkx.cycle_basis()* method to classify all edges into those inside and outside the cycle basis (using the algorithm from Paton [1969]), and assign "dead-ends" and "self-loops" based on appropriate features. Remaining edges are classified as "bridges". Self-loops shorter than 40m are deleted, as these typically indicate a turning circle at the end of a cul-de-sac rather than a separate street.

We also record the path length and end-to-end straight-line distance for each edge.

C.2.3 Node properties

Within each analysis "chunk" of the network, we straightforwardly calculate the nodal degree of each node. Next, we iterate over every node n in the chunk, as follows:

- For all nodes n' within distance r_2 of node n but further than distance r_1 for $(r_1, r_2) \in$ { (0,500m), (500m, 1000m), (1km, 1.5km), (1.5km, 2km), (2km, 2.5km), (2.5km, 3km) }:
  - Calculate the shortest network path (according to unweighted distance, using the Dijkstra [1959] algorithm) between n and n', and calculate the line-of-sight distance between n and n'
- Record the number of nodes n' in each distance band, and the sums of all network distances and of all straight-line distances, and store these values to characterize node n

		Grid	Medieval	Culs-de-sac
Scalar index				
	Sprawl (SNDi)	-0.76	0.91	8.11
Nodes				
	Nodal degree	3.99	3.05	2.19
	Frc deadends	0.00	0.00	0.45
	Frc 1- and 3-degree nodes	0.01	0.95	0.91
Dendricity				
	Frc non-cycle (length)	0.00	0.00	0.40
	Frc bridges (length)	0.00	0.00	0.04
	Frc non-cycle (N edges)	0.00	0.00	0.48
	Frc bridges (N edges)	0.00	0.00	0.07
Circuity				
	Circuity (0–0.5km)	1.24	1.35	2.34
	Circuity (0.5–1km)	1.27	1.33	1.94
	Circuity (1–1.5km)	1.27	1.32	1.64
	Circuity (1.5–2km)	1.27	1.31	1.42
	Circuity (2–2.5km)	1.22	1.21	1.26
	Circuity (2.5–3km)	1.07	1.14	1.10
Sinuosity				
	Curviness	1.00	1.00	1.03

Table C: **Connectivity metrics for street-network**. Each network paradigm is tiled over a large area to minimize edge effects.

D Street-network paradigms

The main text discusses and illustrates the three street-network paradigms that we identify and analyze in order to show how SNDi captures the range of connectivity. Because some components of our connectivity metrics take into account properties of nearby streets 3 km or even further away from the point they characterize, a continuous, or tileable, street network layout is a sensible starting point to understand our component metrics. Thus, each paradigm mimics a piece of a real street network somewhere, but is extended in such a way that it can be continuously tiled.

The simple rectilinear layout of the *grid* paradigm is found in Vancouver, Canada at 49°14'14.3376"N 123°4'52.9824" W. The *medieval* paradigm is modeled after a neighborhood of Istanbul, Turkey at 41°0'2.6928"N 28°55'25.9475" E. The *culs-de-sac* paradigm is based on Sunnyvale, a suburb of San Jose, United States, modeled after a block at 37°21'22.7"N 122°02'21.6"W .

Table C show how both SNDi and its constituent metrics vary across the paradigms. Even though the *medieval* paradigm is not a grid, its overall connectivity as measured by SNDi is much closer to the *grid* than the *culs-de-sac* paradigm. The *medieval* and *grid* paradigms are primarily differentiated by nodal degree and the fraction of 1- and 3-degree nodes; other connectivity measures are comparable.

E Sample grid cells

This section discusses further examples of grid cells across the SNDi distribution. In addition to the US and Indonesia examples in the main text, we show here examples from automatically chosen grid cells close to the 5th, 50th and 95th percentiles of SNDi in the fifteen largest countries by population. The aim is to provide a qualitative validation, using aerial imagery, Google Streetview and other online sources to show how SNDi captures variations in connectivity as well as correlating with other aspects of walkability. Images for each example grid cell are shown in Table D. We discuss only the high-connectivity (5th percentile) and low-connectivity (95th percentile) examples; the median examples are shown for completeness in the table, which provides hyperlinks from each image to aerial imagery and the OpenStreetMap rendering.

E.1 High connectivity (low SNDi)

The high-connectivity examples occur across the spectrum of metropolitan area sizes. The China example shows the Central Business District of Shenyang, a provincial capital of >8 million people; the Japan example is from central Osaka; and the Russian example is the academic district of Moscow. Smaller cities in the examples include Ciudad Constitution, Mexico and Shire, Ethiopia, both of which have a population ∼45,000. In between are mid-sized cities such as Dourados, Brazil (∼210,000) and Bikaner, India (∼640,000).

All the high-connectivity examples, however, are based on a grid or irregular grid, or in the case of Pakistan, a "circular grid" consisting of concentric arcs. They also have a mix of land uses. In the city-center examples, such as China and Japan, medium- to high-rise apartment buildings are adjacent to commercial buildings and services. In the USA, the grid cell includes older residential buildings without off-street parking, more modern high-density residential and mixed-use buildings, office space, and services such as restaurants, churches, and yoga studios. In Indonesia, Bangladesh, the Philippines and Vietnam, the neighborhoods consist of housing and both informal and formal commercial services, from banks to street vendors selling fruit and vegetables.

Walking infrastructure varies among the high-connectivity examples, but tends to be of higher quality than typical for each country. Sidewalks (in most cases, on tree-lined streets) are present in the examples from Brazil, USA, Japan, Russia and Mexico, and intermittently in Bangladesh, Indonesia, the Philippines and Vietnam. (In the remaining countries, the available imagery makes sidewalk presence difficult to verify.)

Many of the examples provide excellent access for vehicles as well as pedestrians. For example, the commercial development in USA, Russia and Mexico is often car-oriented with large off-street parking facilities. However, such car access is not at the expense of sidewalks or other aspects of the walking environment. Indeed, the most limited pedestrian infrastructure occurs in the examples from lower-income countries where the imagery suggests that walking and bicycling are dominant regardless. In Bangladesh, cycle rickshaws appear to be a primary mode. Public transportation, meanwhile, is available in the form of subways, trams or other intra-urban rail services in the examples from China, USA, Russia, and Japan, while the India, Nigeria and Pakistan examples have inter-urban rail service.

E.2 Low connectivity (high SNDi)

The low-connectivity examples generally feature dendritic street networks with frequent culs-de-sac. In many countries, connectivity is intentionally kept low, due to the gated communities in USA

(Orange County, California), Indonesia (a southern suburb of Manila), Pakistan (Rawalpindi), Brazil (Sorocaba, near Sao Paulo), Mexico (Guadalajara), Ethiopia (the outskirts of Addis Ababa) and the Philippines (exurban Manila). In Pakistan, the example shows the planned community of Bahria Town Phase I, which is separated from adjacent development by a 700m access road. In Brazil, the entrance to the gated community features multiple lanes and guard booths, and is reminiscent of an international border crossing. The gated communities can be extremely deep; in the Ethiopian case, the most distant houses are 3 km from the single entrance gate, and there are no commercial services within the compound.

The China (Nanjing) example, meanwhile, does not appear to be gated, but the street design of the planned subdivision is similar to that of a gated community, in common with many of China's superblocks [Kan et al., 2017]. In other examples such as Bangladesh (near Dhaka's airport), Nigeria (the southern suburbs of Ibadan) and Vietnam (Di An, near Ho Chi Minh City), the dendritic street network appears to have grown up more organically, but the effect on restricting connectivity is similar to the planned communities.

In the Bangladesh example, water bodies provide an additional barrier to connectivity. Physical barriers are also evident in the Japan example (in Hiroshima), which is on a steep hillside. Here, ravines separate adjacent residential development, and streets sometimes dead end as the terrain slopes up more steeply.

Especially in the higher- and middle-income countries, the examples are highly car oriented. The USA example features a residential subdivision with large, high-end houses on lots of 1+ acres, few sidewalks, and a Walkscore of 21 out of 100. In several examples, such as the USA and Japan, the streets are dominated by garage doors or parking areas. (The gated communities are likely to be designed in similar ways, but lack street-level imagery on interior streets.) While streets in the Mexico example generally include sidewalks, they run alongside the blank walls of the gated residential subdivisions, and past shopping centres where pedestrians must walk through the parking lot to access the stores.

Table D: **Sample grid cells for the most populous fifteen countries**. Links are given to corresponding Google satellite imagery. A version with all imagery integrated is available online.

	5th percentile (SNDi≈ −0.6)		50th percentile (SNDi≈ 2.7)		95th percentile (SNDi≈ 9.2)	
China	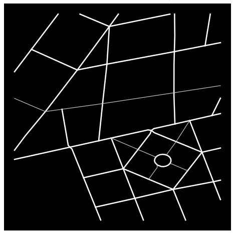	Satellite image	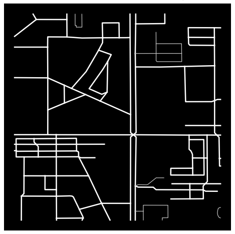	Satellite image	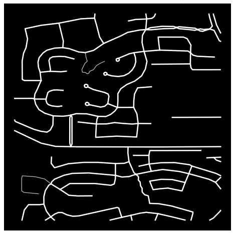	Satellite image
India	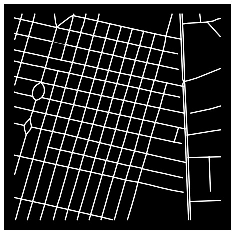	Satellite image	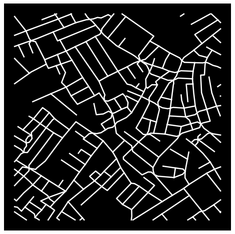	Satellite image	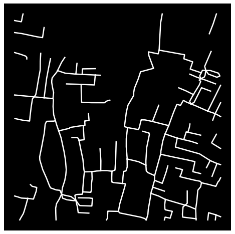	Satellite image
United States of America	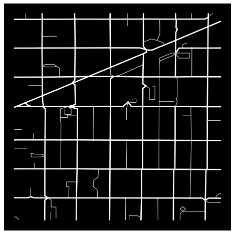	Satellite image	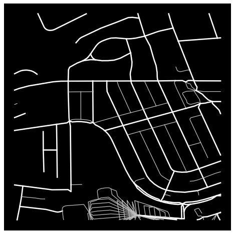	Satellite image	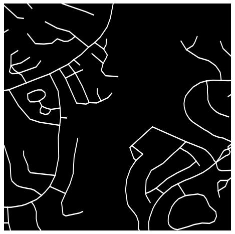	Satellite image
Indonesia	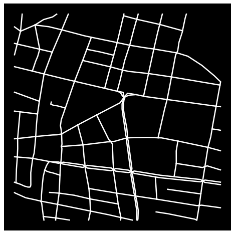	Satellite image	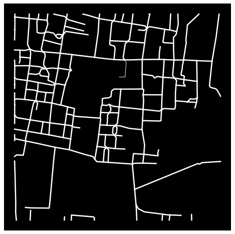	Satellite image	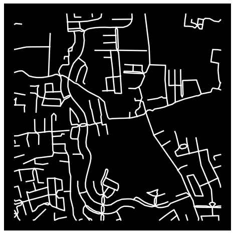	Satellite image
Pakistan	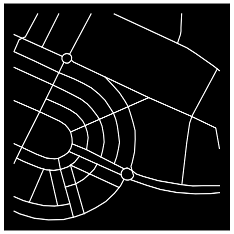	Satellite image	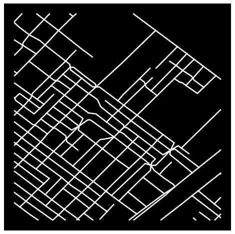	Satellite image	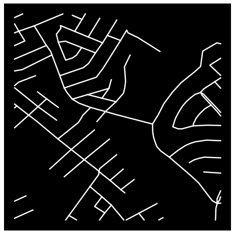	Satellite image

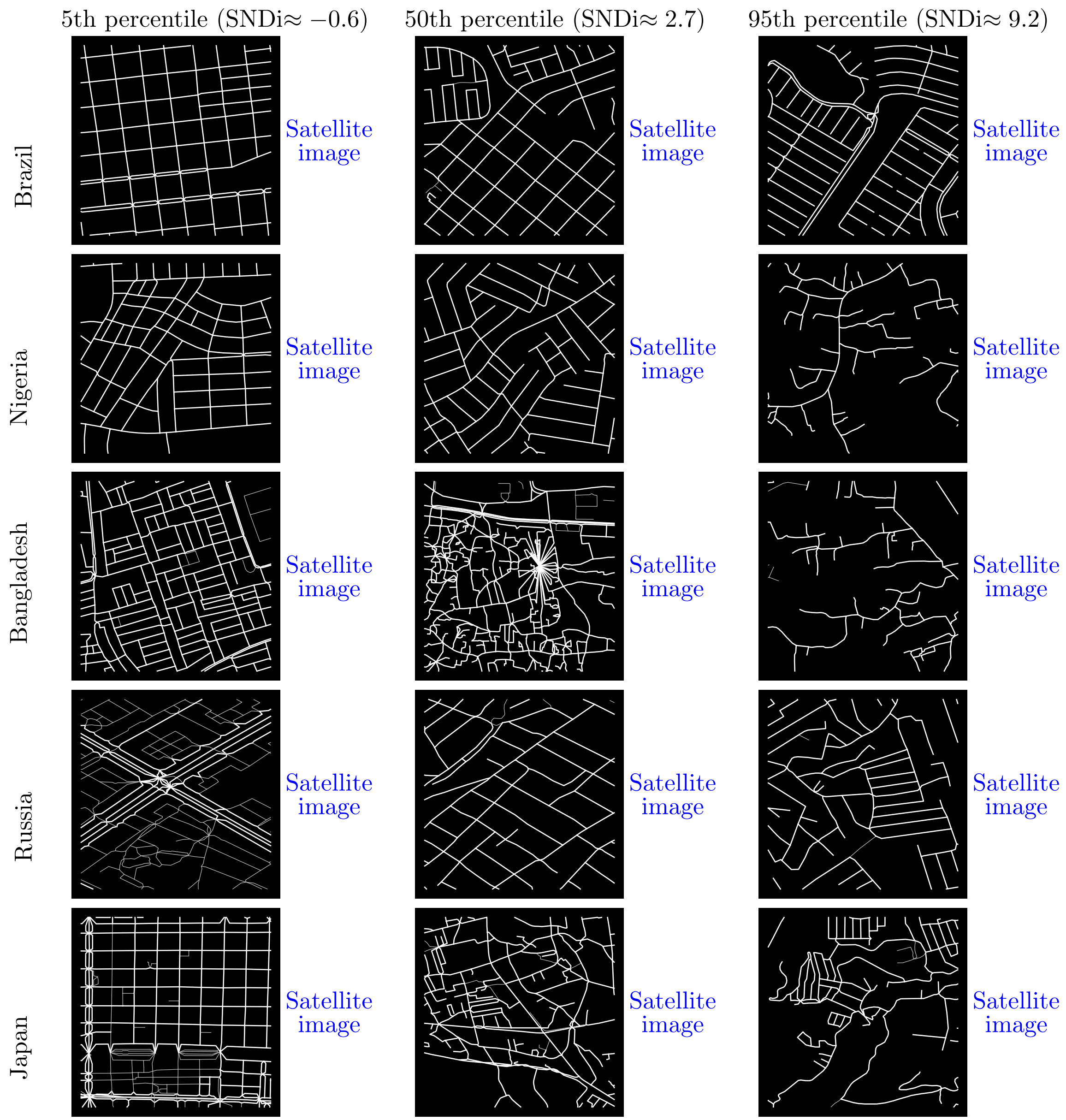

5th percentile (SNDi≈ −0.6)
50th percentile (SNDi≈ 2.7)
95th percentile (SNDi≈ 9.2)
Brazil
Nigeria
Bangladesh
Russia
Japan
Satellite image

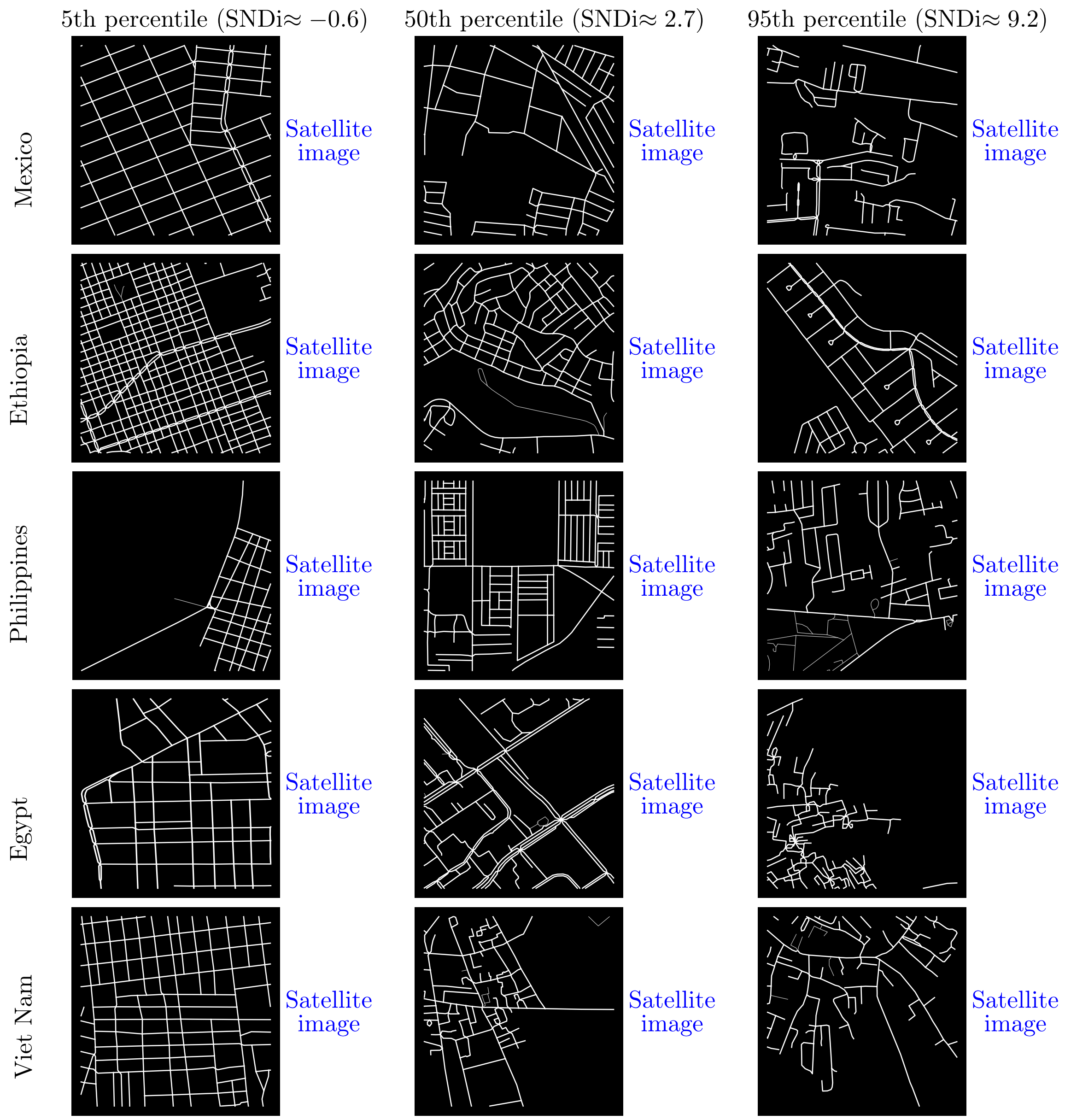
5th percentile (SNDi≈ −0.6)
50th percentile (SNDi≈ 2.7)
95th percentile (SNDi≈ 9.2)
Mexico
Ethiopia
Philippines
Egypt
Viet Nam
Satellite image

F Distributions of SNDi

Figure F shows the distribution of SNDi in the 49 largest countries (by population), and expands on the inset to Figure 5 in the main text. Countries are ordered by mean SNDi.

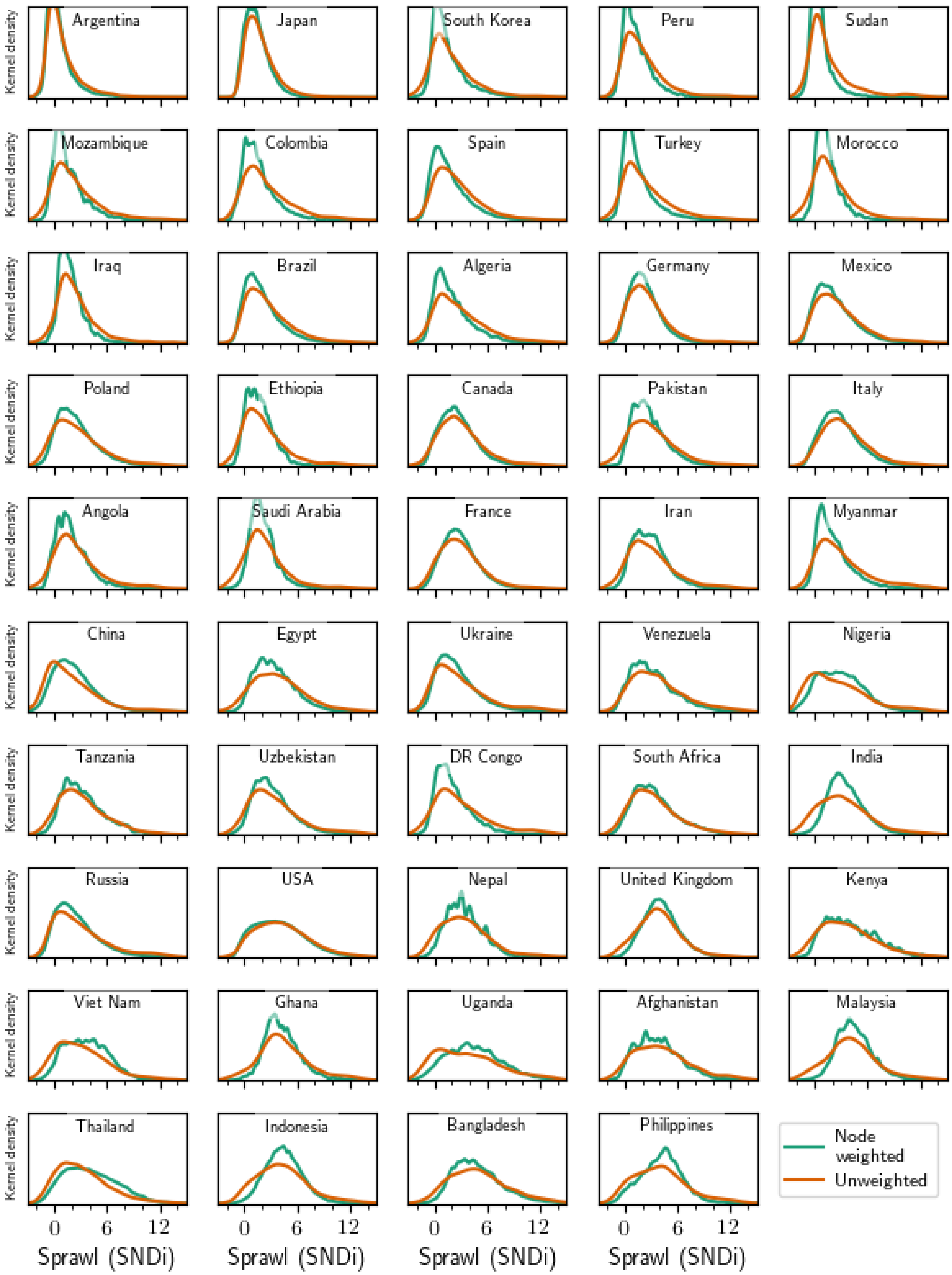

Figure F: **Distribution of SNDi within the 49 largest countries**. The orange lines show unweighted urban grid cells, while the green lines show the distribution weighted by the number of nodes in each grid cell.

G Empirical street-network types (k-means cluster analysis)

We use the *scipy.cluster.vq.kmeans2* procedure in Python from SciPy.org, which provides an unsupervised machine learning algorithm to classify all non-empty urban grid cells into up to ten clusters, or *empirical street-network types*. The clustering is based on eight street-network sprawl metrics which are calculated on each grid cell. Cluster analysis is a descriptive and exploratory tool. As recommended by Jain [2010], we ran the analysis with several values of k and chose the result that was most interpretable. We use k-means rather than other clustering algorithms for computational reasons, and because of its ease of implementation, simplicity, efficiency, and empirical success [Jain, 2010]. Moreover, in contrast to a hierarchical clustering algorithm, a partitional algorithm such as k-means does not impose a hierarchical structure.

We characterize each type by its *centroid*, i.e. the type's mean value of the eight metrics. We then order the types based on the value of SNDi given by each centroid. We ignore two types (6 and 9) that are artefacts of our division into grid cells, for example through consisting almost entirely of dead-ends with the network connections existing in adjacent cells. This yields eight types for the main analysis. The ignored types account for 0.5 per cent of urban nodes.

Figure G portrays the results of the clustering optimization, which minimizes the Euclidean distance between observations and centroids in a space of normalized dimensions. The radial distances of each vertex signify the centroid on each of the eight sprawl metrics for a given type. Given that the number of types is somewhat arbitrary, and the type definitions are influenced by the precise sprawl metrics that are included, the k-means cluster analysis should be seen as a descriptive machine learning technique that helps to identify similarities across world regions in a structured manner. When the polygons in Figure G are concentric, they emphasize that our various connectivity measures tend to co-vary, while the non-concentric cases represent types with characteristics that may differentiate themselves from other types with similar average values of SNDi.

The types have an intuitive relationship to the paradigms discussed in the main text. Our *grid* and *medieval* paradigms map to Types A (grid) and B (degree-3) respectively. Our *culs-de-sac* paradigm maps most closely to Type H (circuitous), but is also close to Type F (dead ends).

The 12 grid cells that are closest to each type centroid (including types 6 and 9), and include at least 25 nodes, are shown in Section M.3 on pages 52–63. The distributions of the types are shown by country (Figure H) and city (Figure I and Figure J); these figures are similar to those in the main text, but show results for a more extensive set of geographies.

Figure K shows that the types have a strong relationship with transportation outcomes — household vehicle ownership and commute by walking — in the United States.

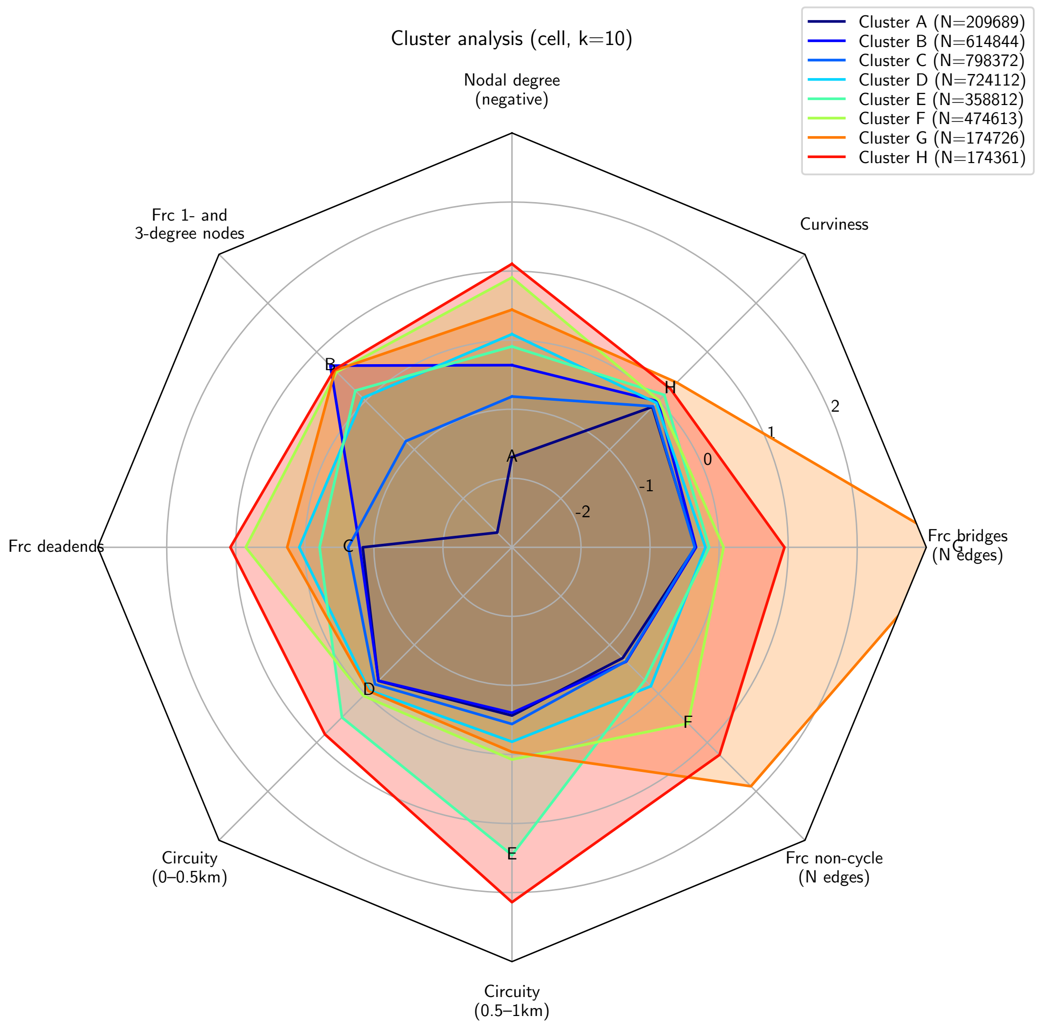

Figure G: **Radar plot of k-means cluster analysis**

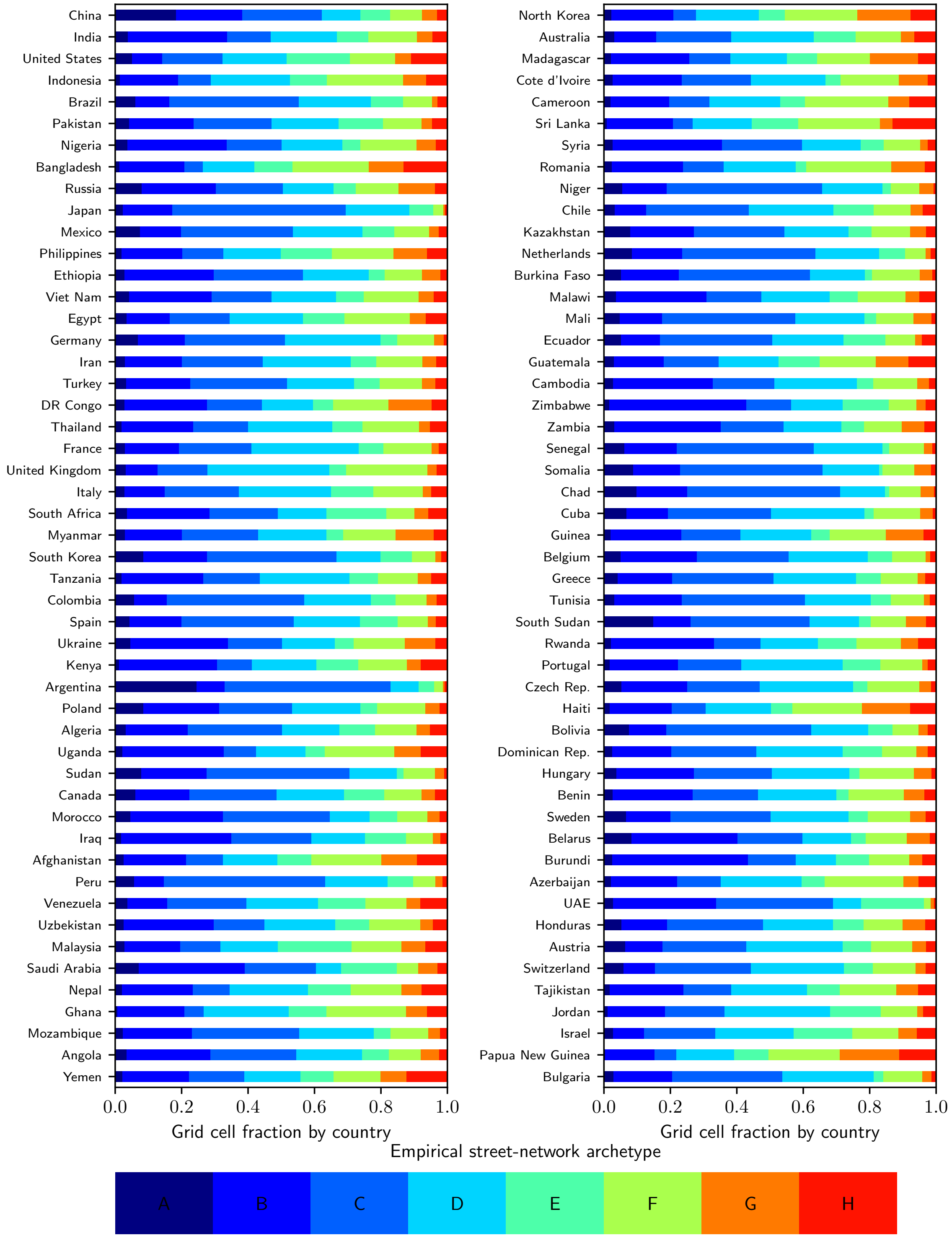

Figure H: **Distribution of empirical street-network types within 100 largest countries**

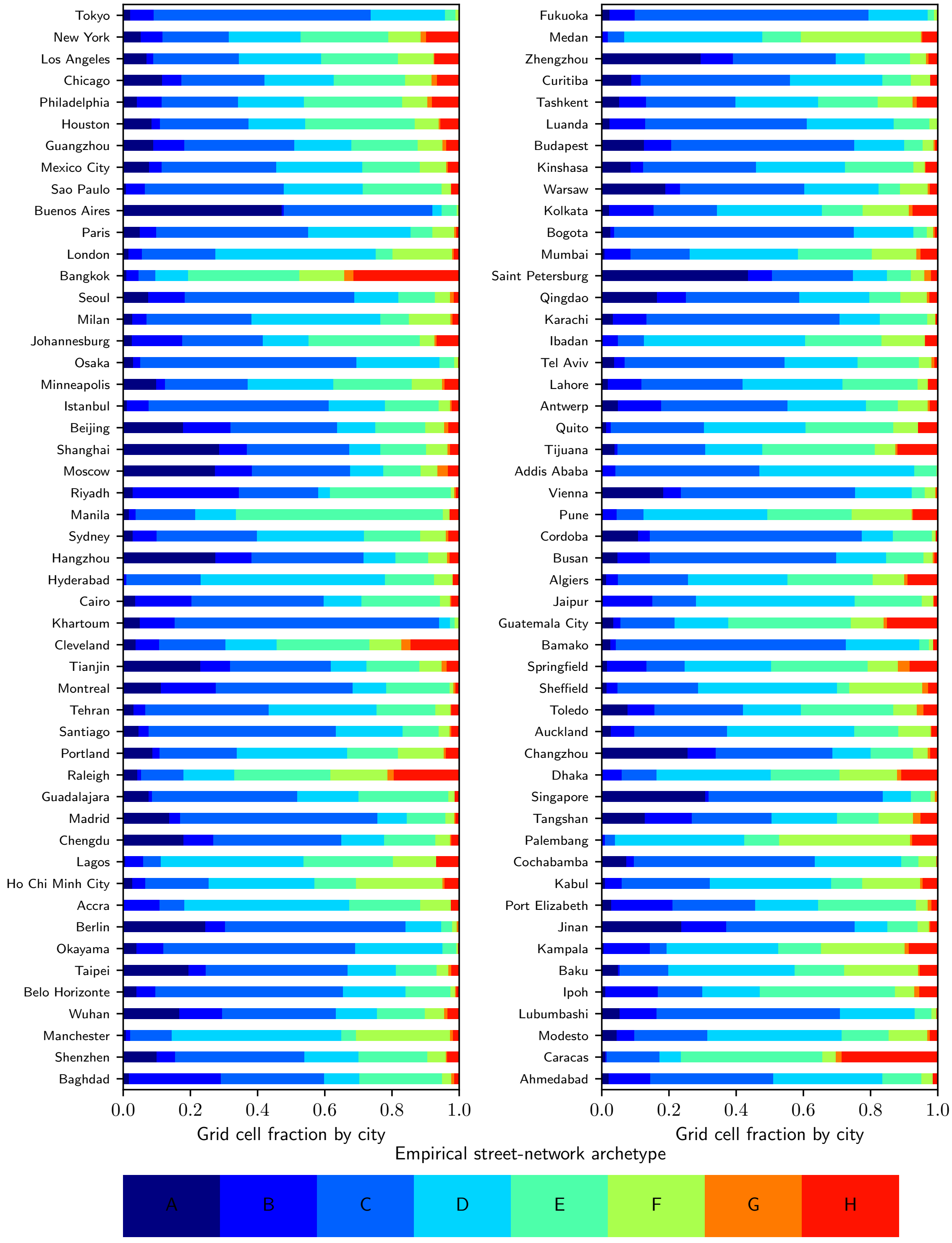

Figure I: **Distribution of empirical street-network types within large cities**. Cities are defined in the Atlas of Urban Expansion (Section §B.3.2), and are sorted by the length of the OSM street network. The figure continues in Figure J.

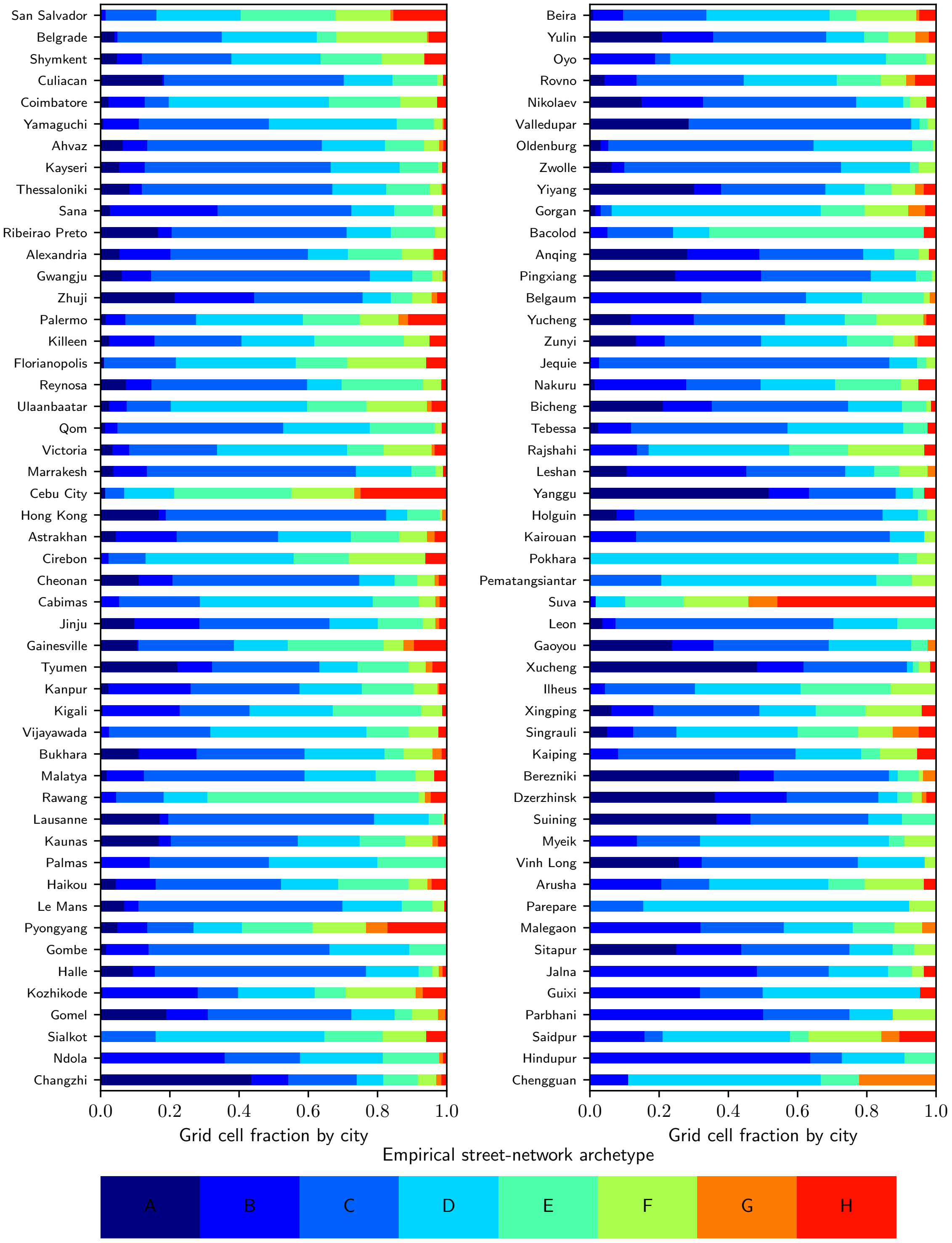

Figure J: **Distribution of empirical street-network types within large cities** (continued from Figure I). Cities are defined in the Atlas of Urban Expansion (Section §B.3.2)

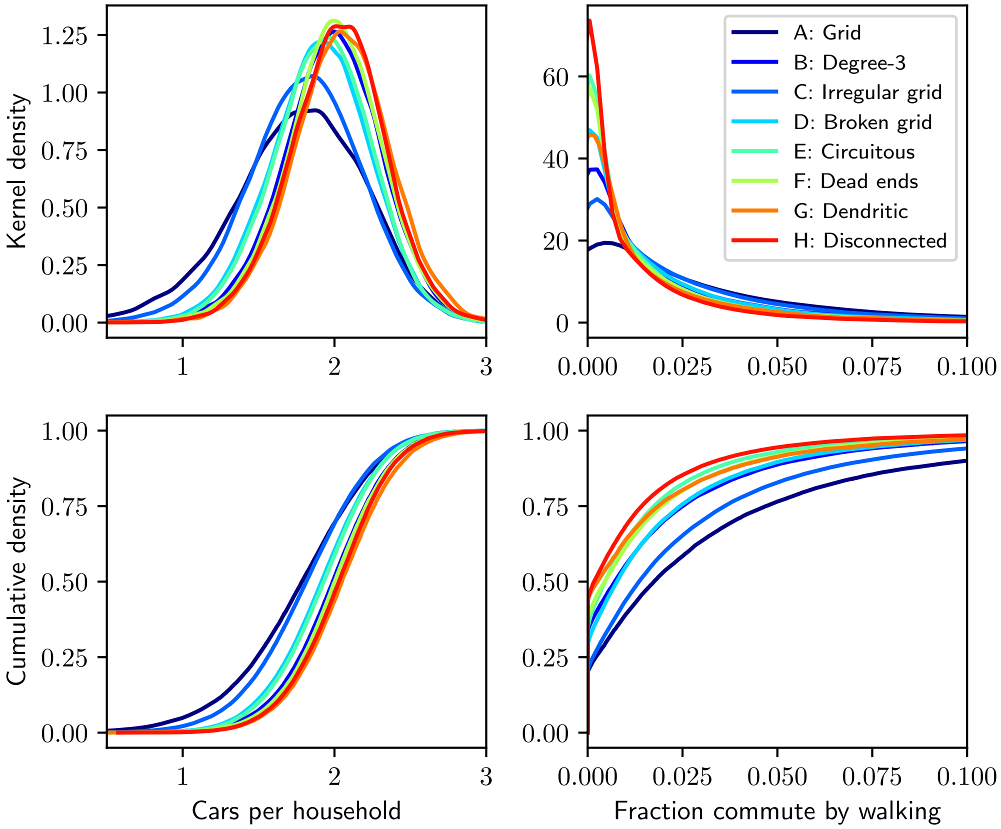

Figure K: **Distribution of vehicle ownership and walking across empirical street-network types, United States**. The upper panels show the kernel density, while the lower panels show the cumulative density.

	Cars per household			Fraction walking			Cars per household
	(1)	(2)	(3)	(4)	(5)	(6)	(7)
SNDi	**.35**†	**.25**†	**.14**†	**−.25**†	**−.23**†	**−.11**†	**.43**†
	(.002)	(.002)	(.003)	(.002)	(.002)	(.004)	(.067)
Nodal degree ×(−1)			**.14**†			**−.14**†	
			(.004)			(.006)	
log(density)		**−.42**†	**−.39**†		**.091**†	**.066**†	**−.43**†
		(.002)	(.002)		(.003)	(.003)	(.085)
country fixed effects	✓	✓	✓	✓	✓	✓	✓
metro Japan							✓
R^2_{adjusted}	.276	.438	.442	.063	.071	.075	.606
N	231800	231800	231800	214800	214800	214800	165

Significance: **0.1%**† **1%**⋆ ***5%*** *10%*+

Table E: **Regressions of vehicle ownership and walking.** Coefficients shown are standardized (beta) coefficients, with robust standard errors in parentheses. Constant terms are not shown.

H Automobility regressions

The main text reports regressions of automobility (vehicle ownership and commute mode share by walking) on SNDi, density and nodal degree. The results also hold in three of the four countries — the United States, France and New Zealand — when analyzed separately, but not in Japan. Most Japanese municipalities are small and rural, and street-network connectivity does not appear to meaningfully influence car ownership in these places. Restricting the regression to wards and municipalities in the three metropolitan prefectures (Tokyo, Kyoto and Osaka) generates a similar result to the main regressions for the other countries, but the discrepancy suggests avenues for more in-depth study. Table E repeats the results from the main text, along with the metropolitan Japan-only regression (model 7).

I Sensitivity analysis

We consider below several possible limits to the external validity or accuracy of our work. These relate to our use of imperfectly-complete OpenStreetMap data, our treatment of pedestrian and bicycle paths, our choices in estimating a principal component, and our choice to treat intersections within 20 m of each other as a single intersection.

I.1 Incompleteness tests

We calculate our full suite of connectivity metrics at varying levels of completeness for eight countries, which are randomly selected from the countries where the current OpenStreetMap (OSM) database is effectively complete according to our analysis in Barrington-Leigh and Millard-Ball [2017]. For each selected country, we use older versions of the OSM database corresponding to road network completeness of ∼50%, ∼60%, ∼70%, ∼80%, ∼90%, ∼95% and 100%. The equivalent date varies by country; for example, 50% completeness in Germany corresponds to August 14, 2008, while in the United Arab Emirates it corresponds to July 24, 2012.

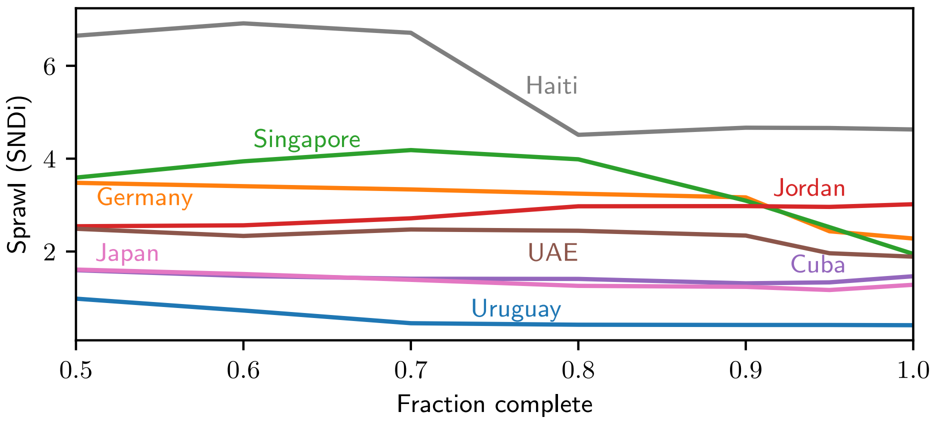

Figure L: **Trends in SNDi, calculated with different levels of completeness of the OSM database**

Figure L plots SNDi as a function of the completeness of the OSM database. Even at 50% completeness, SNDi hardly differs from the 100% complete value, except in Haiti and Singapore. In Haiti, part of the reason is that a major redaction of ways occurred circa 2010, when data that did not meet OSM license conditions was purged from the OSM database. As a result, some key network links are unavailable in the OSM history, and thus SNDi for completeness levels <=0.7 is inflated through greater circuity and other measures of sprawl.

Moreover, the ordinal ranking of the countries shows minimal change. We conclude that even though for many countries, OSM is still missing many streets, this issue is unlikely to affect our qualitative findings. Note that contributions to OSM over this period primarily reflect corrections to incomplete mapping, rather than the construction of new roads. The abscissa therefore does not correspond to the time dimension in our main analysis.

I.2 PCA weighting

Our PCA coefficients (see Section §A.6) are estimated using ∼126,000 regions representing the highest-resolution GADM (see Section §B.3.1) geographies available at each location. The geographic size of these regions varies greatly due to the different scales of administrative regions in GADM for each country. Correspondingly, the number of nodes and length of road also varies greatly across the regions used for the estimate. We therefore experimented with weighting the regions by the number of nodes they contained or the population they included (according to Landscan data; see Section §B.3.3) during the PCA estimate. We find that estimated loading coefficients for PCA vary little with different choices of weighting scheme.

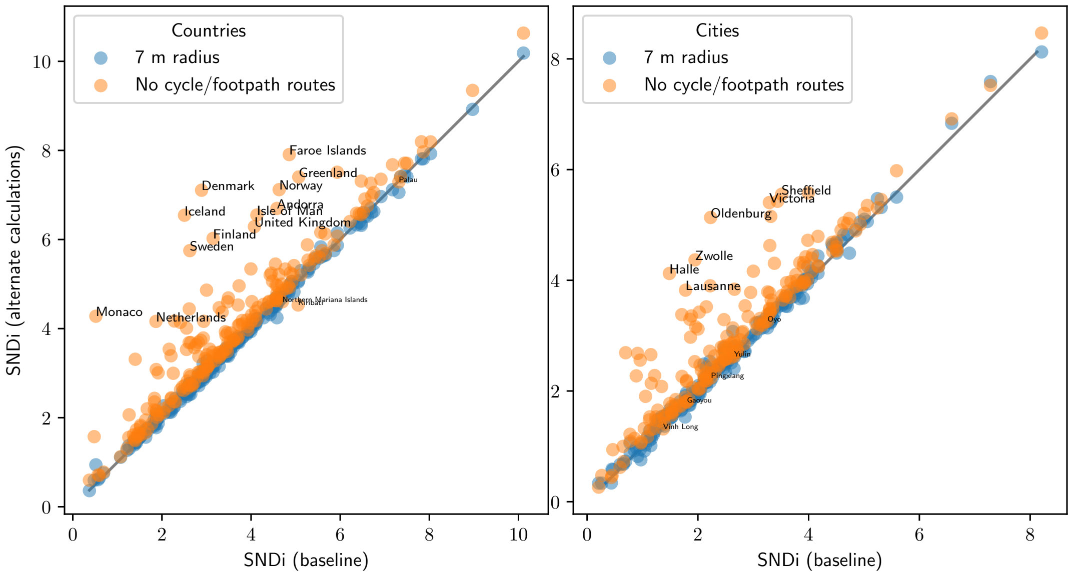

Figure M: **Robustness of SNDi to alternate assumptions**.

I.3 Buffer radius used to separate nodes/junctions

We reran our entire analysis using a radius of 7 m for merging intersections into nodes in our network representation. Aggregate findings are nearly identical to the 10 m version used in our primary analysis. The blue markers in Figure M all lie almost precisely on the 45° line for both a country- and city-level analysis, indicating that there is no meaningful difference between analyses using different buffer radii. The PCA coefficients are also virtually identical, regardless of whether a 10 m or 7 m radius is used (Figure N).

I.4 Exclusion of walking and cycling paths

Our main results aggregate the properties of only those edges and nodes that are accessible by motor vehicle. However, walking and cycling paths, as well as service roads such as driveways, are considered when calculating connectivity (see Section §B.1). For example, two adjacent culs-de-sac that are connected by a pedestrian path would not be considered deadends. In order to test whether our findings are sensitive to this choice, we repeated all our calculations using an alternative formulation in which walking paths, cycling paths, and service roads are entirely ignored, including in the calculation of connectivity. We find that our PCA coefficients for SNDi remain the same under this alternative (Figure N). We also find closely similar values of SNDi for city and country aggregates for the large majority of cities and countries (orange markers in Figure M). Several countries and cities, particularly in the UK, Scandinavia and other parts of Europe, show substantially higher SNDi when the extra paths are completely excluded, indicating that such paths make a major contribution to the connectivity of the street network.

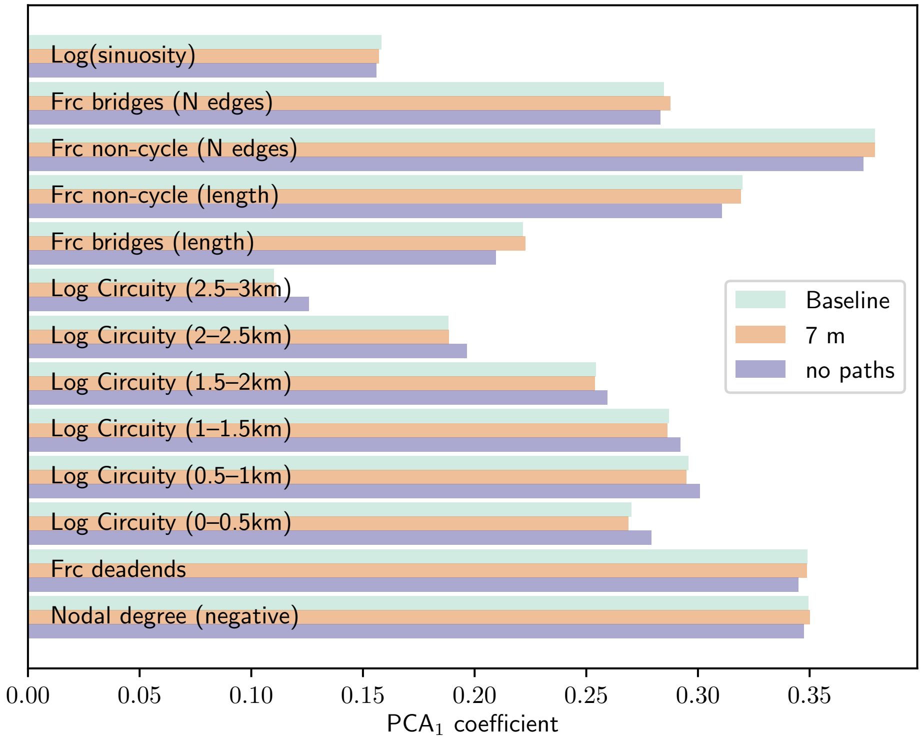

Figure N: **Robustness of principal component coefficients to alternate assumptions**. Coefficients defining the first principal component are shown for each of three different computations. The two alternative cases give similar results to our baseline case, which uses a 10 m buffer radius and considers secondary paths and service roads when calculating connectivity between points on a road.

J Data release

All data used for this work, with the exception of some Japanese car registration data, are open. Freely available sources are given in Sections B.1 and B.3. See Section §K for instructions on reproducing the entirety of our work (including server-setup, data preparation, computation, and analysis) using our open-source software which itself leverages exclusively open-source tools.

In addition, an interactive map interface to our high-resolution results, with links to data downloads, is available at https://sprawlmap.org.

K Open-source code

Our code to reproduce the data and analysis is released under the GNU General Public License v3.0 as an open source project, permanently available at:

`https://alum.mit.edu/www/cpbl/publications/2019-PLOS-sprawl/code`

That site includes the following description of the code:
`https://gitlab.com/cpbl/global-sprawl-2019/blob/master/README.md`

L Citation and contact

For any use of the data or code, please cite the main paper.

For further questions, please contact:
Christopher Barrington-Leigh, McGill University
Adam Millard-Ball, University of California, Santa Cruz

References

Shlomo Angel, Alejandro M Blei, Daniel L Civco, and Jason Parent. *Atlas of urban expansion*. Lincoln Institute of Land Policy, Cambridge, MA, 2012. URL `http://atlasofurbanexpansion.org`.

Shlomo Angel, Alejandro M. Blei, Jason Parent, Patrick Lamson-Hall, Nicolás Galarza Sánchez, with Daniel L. Civco, Rachel Qian Lei, and Kevin Thom. *Atlas of Urban Expansion — 2016 Edition: Volume 1: Areas and Densities*. NYU Urban Expansion Program at New York University, UN-Habitat, and the Lincoln Institute of Land Policy, 10 2016. URL `http://www.lincolninst.edu/publications/other/atlas-urban-expansion-2016-edition`.

Christopher Barrington-Leigh and Adam Millard-Ball. The world's user-generated road map is more than 80% complete. *PLOS ONE*, 12(8):1–20, 08 2017. doi: 10.1371/journal.pone.0180698. URL `https://doi.org/10.1371/journal.pone.0180698`.

Geoff Boeing. OSMnx: New methods for acquiring, constructing, analyzing, and visualizing complex street networks. *Computers, Environment and Urban Systems*, 65(Supplement C):126 – 139, 2017. ISSN 0198-9715. doi: https://doi.org/10.1016/j.compenvurbsys.2017.05.004. URL `http://www.sciencedirect.com/science/article/pii/S0198971516303970`.

Geoff Boeing. A multi-scale analysis of 27,000 urban street networks: Every US city, town, urbanized area, and Zillow neighborhood. *Environment and Planning B: Urban Analytics and City Science*, Online First, 2018. ISSN 2399-8083, 2399-8091. doi: 10.1177/2399808318784595. URL http://journals.sagepub.com/doi/10.1177/2399808318784595.

Barney Cohen. Urbanization in developing countries: Current trends, future projections, and key challenges for sustainability. *Technology in Society*, 28(1):63 – 80, 2006. ISSN 0160-791X. doi: https://doi.org/10.1016/j.techsoc.2005.10.005. URL http://www.sciencedirect.com/science/article/pii/S0160791X05000588.

E. W. Dijkstra. A note on two problems in connexion with graphs. *Numerische Mathematik*, 1(1):269–271, Dec 1959. ISSN 0945-3245. doi: 10.1007/BF01386390. URL https://doi.org/10.1007/BF01386390.

Eric Eidlin. What density doesn't tell us about sprawl. *Access*, 37(Fall 2010):2–9, 2010. URL http://www.accessmagazine.org/wp-content/uploads/sites/7/2016/01/access37sprawl.pdf.

David J. Giacomin and David M. Levinson. Road network circuity in metropolitan areas. *Environment & Planning B: Planning & Design*, 42:1040–1053, 2015. doi: 10.1068/b130131p.

Anil K. Jain. Data clustering: 50 years beyond k-means. *Pattern Recognition Letters*, 31(8):651 – 666, 2010. ISSN 0167-8655. doi: https://doi.org/10.1016/j.patrec.2009.09.011. URL http://www.sciencedirect.com/science/article/pii/S0167865509002323. Award winning papers from the 19th International Conference on Pattern Recognition (ICPR).

Jamal Jokar Arsanjani, Alexander Zipf, Peter Mooney, and Marco Helbich. An introduction to OpenStreetMap in Geographic Information Science: Experiences, research, and applications. In Jamal Jokar Arsanjani, Alexander Zipf, Peter Mooney, and Marco Helbich, editors, *OpenStreetMap in GIScience*, Lecture Notes in Geoinformation and Cartography, pages 1–15. Springer International Publishing, 2015. ISBN 978-3-319-14279-1. doi: 10.1007/978-3-319-14280-7_1. URL http://dx.doi.org/10.1007/978-3-319-14280-7_1.

Har Ye Kan, Ann Forsyth, and Peter Rowe. Redesigning China's superblock neighbourhoods: policies, opportunities and challenges. *Journal of Urban Design*, 22(6):757–777, 2017. doi: 10.1080/13574809.2017.1337493. URL https://doi.org/10.1080/13574809.2017.1337493.

Oak Ridge National Laboratory. Landscan 2012, 2013. URL http://web.ornl.gov/sci/landscan/.

Stephen Marshall, Jorge Gil, Karl Kropf, Martin Tomko, and Lucas Figueiredo. Street network studies: from networks to models and their representations. *Networks and Spatial Economics*, Nov 2018. ISSN 1572-9427. doi: 10.1007/s11067-018-9427-9. URL https://doi.org/10.1007/s11067-018-9427-9.

OpenStreetMap. OpenStreetMap Statistics, 2018. URL http://www.openstreetmap.org/stats/data_stats.html.

Pavithra Parthasarathi, Hartwig Hochmair, and David Levinson. Street network structure and household activity spaces. *Urban Studies*, 52(6):1090–1112, July 2015. ISSN 0042-0980. doi: 10.1177/0042098014537956. URL http://usj.sagepub.com/content/early/2014/07/02/0042098014537956.abstract.

Keith Paton. An algorithm for finding a fundamental set of cycles of a graph. *Communications of the ACM*, 12(9):514–518, 1969.

Martino Pesaresi, Guo Huadong, Xavier Blaes, Daniele Ehrlich, Stefano Ferri, Lionel Gueguen, Matina Halkia, Mayeul Kauffmann, Thomas Kemper, Linlin Lu, et al. A global human settlement layer from optical HR/VHR RS data: concept and first results. *IEEE Journal of Selected Topics in Applied Earth Observations and Remote Sensing*, 6(5):2102–2131, 2013.

David Potere and Annemarie Schneider. A critical look at representations of urban areas in global maps. *GeoJournal*, 69(1):55–80, 2007. doi: 10.1007/s10708-007-9102-z. URL http://dx.doi.org/10.1007/s10708-007-9102-z.

Michael Southworth and Eran Ben-Joseph. *Streets and the Shaping of Towns and Cities*. Island Press, Washington, D.C., 2nd edition, 2003.

United Nations. 2013 Demographic Yearbook. Sixty-fourth issue. Technical report, UN Department of Economic and Social Affairs, New York, 2014. URL http://unstats.un.org/unsd/demographic/products/dyb/dyb2014.htm.

Yuyu Zhou, Steven J Smith, Kaiguang Zhao, Marc Imhoff, Allison Thomson, Ben Bond-Lamberty, Ghassem R Asrar, Xuesong Zhang, Chunyang He, and Christopher D Elvidge. A global map of urban extent from nightlights. *Environmental Research Letters*, 10(5):054011, 2015. URL http://stacks.iop.org/1748-9326/10/i=5/a=054011.

M Results

The sections which follow contain more detailed tabular and graphical renditions of our findings. In addition, extensive online visualizations of our results are available at:
https://alum.mit.edu/www/cpbl/publications/2019-PLOS-sprawl
including an interactive map interface to our high-resolution results at:
https://sprawlmap.org.

M.1 Countries ranked by urban road network sprawl (SNDi) of total road stock

Table F: **Countries listed in order of the connectivity (SNDi) of their total stock of urban streets**. Countries with large (>20M) population are listed in bold.

	Sprawl (SNDi)	Nodal degree	Frc non-cycle (length)	Log Circuity (0.5–1km)	N(nodes)
Virgin Islands (U.S.)	7.3	2.5	0.3	0.3	5.3k
Liberia	6.9	2.5	0.5	0.2	22k
Brunei Darussalam	6.7	2.4	0.3	0.2	14k
Puerto Rico	6.5	2.5	0.3	0.2	120k
Papua New Guinea	6.5	2.6	0.4	0.2	6.2k
Barbados	6.4	2.4	0.3	0.2	13k
Kosovo	6.4	2.3	0.4	0.2	41k
Trinidad and Tobago	6.2	2.5	0.3	0.2	30k
Ireland	5.9	2.6	0.3	0.2	120k
Gabon	5.9	2.5	0.3	0.2	15k
Sierra Leone	5.7	2.6	0.3	0.2	25k
Jamaica	5.6	2.6	0.2	0.2	33k
**Sri Lanka**	5.5	2.5	0.2	0.2	150k
**Philippines**	5.4	2.8	0.3	0.2	500k
**Korea, Dem. People's Rep.**	5.4	2.6	0.3	0.2	58k
**Bangladesh**	5.3	2.6	0.3	0.2	160k
**Madagascar**	5.3	2.7	0.5	0.2	39k
**Indonesia**	5.1	2.7	0.3	0.2	1.6M
Albania	4.9	2.6	0.3	0.2	41k
**Thailand**	4.9	2.7	0.2	0.2	920k
**Malaysia**	4.9	2.8	0.2	0.2	420k
Bahamas, The	4.8	2.7	0.2	0.2	9.2k
**Afghanistan**	4.8	2.7	0.3	0.2	97k
Bosnia and Herzegovina	4.8	2.6	0.2	0.2	42k
Costa Rica	4.7	2.7	0.2	0.2	61k
**Uganda**	4.7	2.6	0.2	0.2	120k
Norway	4.6	2.8	0.2	0.2	170k
Haiti	4.6	2.7	0.3	0.2	40k
Azerbaijan	4.6	2.6	0.2	0.2	130k
Macedonia, FYR	4.6	2.6	0.3	0.2	36k
**Ghana**	4.6	2.7	0.2	0.2	130k
Panama	4.6	2.8	0.2	0.2	37k
Guatemala	4.6	2.9	0.3	0.2	110k
Slovenia	4.5	2.6	0.2	0.2	61k
**Cameroon**	4.5	2.7	0.2	0.2	150k
West Bank and Gaza	4.5	2.6	0.2	0.2	55k
Montenegro	4.4	2.7	0.2	0.2	8.6k
**Vietnam**	4.4	2.8	0.2	0.2	290k
El Salvador	4.3	2.8	0.2	0.2	66k

	Sprawl (SNDi)	Nodal degree	Frc non-cycle (length)	Log Circuity (0.5–1km)	N(nodes)
Cyprus	4.2	2.7	0.2	0.2	41k
Georgia	4.1	2.7	0.2	0.2	95k
**Kenya**	4.1	2.7	0.2	0.2	58k
**United Kingdom**	4.1	2.7	0.2	0.2	1.6M
Tajikistan	4.0	2.8	0.2	0.2	33k
Guinea	4.0	2.8	0.3	0.2	52k
**Nepal**	4.0	2.7	0.2	0.2	63k
Lao PDR	4.0	2.7	0.2	0.2	18k
New Zealand	4.0	2.8	0.2	0.2	96k
Lebanon	3.9	2.7	0.2	0.2	45k
**United States**	3.9	2.9	0.2	0.2	11M
Armenia	3.8	2.8	0.2	0.2	46k
Serbia	3.8	2.8	0.2	0.2	100k
Guyana	3.7	2.9	0.2	0.2	10k
Croatia	3.7	2.7	0.2	0.2	87k
Mongolia	3.7	2.8	0.2	0.2	27k
**Russian Federation**	3.7	2.9	0.3	0.2	1.9M
Malawi	3.7	2.8	0.1	0.2	58k
**India**	3.7	2.8	0.2	0.2	2.4M
Rwanda	3.7	2.8	0.1	0.2	15k
Central African Republic	3.6	3.0	0.4	0.1	12k
**South Africa**	3.6	2.9	0.1	0.2	660k
**Congo, Dem. Rep.**	3.6	2.9	0.3	0.2	290k
Curacao	3.5	2.8	0.1	0.2	7k
Slovak Republic	3.5	2.8	0.2	0.1	110k
**Uzbekistan**	3.5	2.8	0.2	0.2	210k
**Nigeria**	3.5	2.8	0.2	0.2	1.3M
**Australia**	3.5	2.9	0.1	0.2	710k
**Tanzania**	3.5	2.8	0.2	0.2	110k
**Romania**	3.4	2.7	0.2	0.1	370k
**Venezuela, RB**	3.4	3.0	0.2	0.2	290k
**Ukraine**	3.4	2.8	0.2	0.2	760k
Oman	3.4	2.8	0.1	0.2	53k
Honduras	3.4	3.1	0.3	0.2	75k
**Egypt, Arab Rep.**	3.4	2.9	0.2	0.2	460k
Lesotho	3.4	2.8	0.2	0.1	43k
Cambodia	3.3	2.9	0.1	0.2	41k
Zambia	3.3	2.9	0.3	0.2	73k
Burundi	3.3	2.9	0.1	0.2	20k
**Cote d'Ivoire**	3.3	2.9	0.2	0.2	79k
**Yemen, Rep.**	3.3	3.0	0.3	0.1	48k
Israel	3.3	2.9	0.2	0.2	99k

	Sprawl (SNDi)	Nodal degree	Frc non-cycle (length)	Log Circuity (0.5–1km)	N(nodes)
Suriname	3.2	2.9	0.2	0.2	9.4k
Kyrgyz Republic	3.2	2.9	0.2	0.2	52k
Finland	3.1	2.9	0.2	0.2	160k
Zimbabwe	3.1	2.9	0.1	0.2	66k
**China**	3.1	3.0	0.2	0.2	1.9M
Portugal	3.1	2.9	0.1	0.2	390k
**Myanmar**	3.0	2.9	0.2	0.1	180k
Mauritius	3.0	2.8	0.1	0.2	21k
Kazakhstan	3.0	3.0	0.2	0.2	150k
Jordan	3.0	2.9	0.1	0.2	93k
**Iran, Islamic Rep.**	3.0	2.9	0.2	0.2	950k
Kuwait	3.0	3.2	0.1	0.2	37k
Austria	2.9	2.9	0.1	0.2	260k
**France**	2.9	2.9	0.1	0.2	2.2M
**Saudi Arabia**	2.9	3.1	0.2	0.2	420k
**Angola**	2.9	3.0	0.3	0.1	150k
Qatar	2.9	3.0	0.1	0.2	33k
Denmark	2.9	2.9	0.1	0.1	240k
Lithuania	2.9	2.9	0.1	0.2	57k
Nicaragua	2.9	3.0	0.2	0.1	49k
**Italy**	2.8	2.9	0.1	0.1	1.5M
Belarus	2.8	2.9	0.2	0.2	160k
**Pakistan**	2.8	3.0	0.1	0.2	250k
Cabo Verde	2.8	3.1	0.1	0.2	5.7k
Namibia	2.8	3.0	0.1	0.2	18k
**Canada**	2.7	3.0	0.1	0.2	720k
Congo, Rep.	2.7	3.1	0.2	0.2	53k
**Ethiopia**	2.7	3.0	0.2	0.1	190k
Dominican Republic	2.7	3.0	0.1	0.2	110k
**Poland**	2.7	2.9	0.1	0.2	530k
Moldova	2.6	2.9	0.2	0.2	88k
Sweden	2.6	3.0	0.1	0.1	300k
Togo	2.6	2.9	0.2	0.1	53k
**Mexico**	2.6	3.1	0.2	0.2	1.9M
Switzerland	2.6	3.0	0.1	0.1	210k
Estonia	2.6	3.0	0.1	0.2	33k
Chile	2.6	3.0	0.1	0.2	320k
Eritrea	2.6	3.1	0.3	0.1	18k
Czech Republic	2.6	2.9	0.1	0.1	270k
Latvia	2.6	2.9	0.1	0.2	46k
Iceland	2.5	3.0	0.1	0.1	12k
Benin	2.5	2.9	0.2	0.1	68k

	Sprawl (SNDi)	Nodal degree	Frc non-cycle (length)	Log Circuity (0.5–1km)	N(nodes)
Bahrain	2.5	3.0	0.1	0.2	30k
Luxembourg	2.4	3.0	0.1	0.1	16k
Turkmenistan	2.4	3.1	0.1	0.2	26k
Libya	2.3	3.0	0.1	0.2	86k
**Germany**	2.3	3.0	0.1	0.1	2.3M
**Algeria**	2.3	3.0	0.1	0.1	410k
Hungary	2.3	2.9	0.1	0.1	230k
Ecuador	2.2	3.1	0.1	0.2	210k
Botswana	2.2	3.0	0.1	0.1	71k
Belize	2.2	3.0	0.1	0.1	9k
Belgium	2.2	3.0	0.1	0.1	290k
**Brazil**	2.1	3.1	0.1	0.2	3.4M
**Iraq**	2.0	3.1	0.1	0.2	320k
Tunisia	1.9	3.0	0.1	0.1	150k
Singapore	1.9	3.3	0.1	0.2	18k
**Turkey**	1.9	3.1	0.1	0.1	1.1M
**Morocco**	1.9	3.1	0.1	0.1	280k
South Sudan	1.9	3.3	0.3	0.1	21k
United Arab Emirates	1.9	3.1	0.0	0.2	90k
Netherlands	1.9	3.1	0.1	0.1	500k
Bulgaria	1.9	3.0	0.1	0.1	230k
**Spain**	1.8	3.1	0.1	0.1	1.4M
**Colombia**	1.8	3.2	0.1	0.1	350k
**Mozambique**	1.8	3.0	0.1	0.1	130k
**Syrian Arab Republic**	1.7	3.0	0.1	0.1	190k
Malta	1.7	3.1	0.1	0.1	13k
**Sudan**	1.6	3.3	0.3	0.1	320k
Mali	1.5	3.2	0.2	0.1	89k
**Peru**	1.5	3.2	0.1	0.1	280k
Greece	1.5	3.1	0.1	0.1	380k
Burkina Faso	1.5	3.1	0.2	0.1	74k
Bolivia	1.5	3.2	0.1	0.1	170k
Senegal	1.5	3.2	0.1	0.1	150k
Cuba	1.4	3.1	0.1	0.1	100k
Niger	1.4	3.2	0.2	0.1	46k
Hong Kong SAR, China	1.4	3.3	0.1	0.1	11k
Gambia, The	1.4	3.1	0.2	0.1	19k
Paraguay	1.4	3.2	0.1	0.1	89k
**Korea, Rep.**	1.4	3.2	0.1	0.1	370k
Taiwan, China	1.3	3.2	0.1	0.1	160k
**Japan**	1.3	3.1	0.1	0.1	4M
Chad	1.2	3.3	0.2	0.1	34k

	Sprawl (SNDi)	Nodal degree	Frc non-cycle (length)	Log Circuity (0.5–1km)	N(nodes)
Somalia	1.1	3.3	0.2	0.1	60k
Maldives	0.7	3.0	0.2	0.1	7k
Mauritania	0.6	3.3	0.1	0.1	26k
**Argentina**	0.6	3.4	0.0	0.1	820k
Uruguay	0.4	3.4	0.0	0.1	66k

M.2 Cities ranked by urban road network sprawl (SNDi) of total road stock

Table G: **Cities listed in order of the connectivity (SNDi) of their total stock of urban streets**.

	Sprawl (SNDi)	Nodal degree	Frc non-cycle (length)	Log Circuity (0.5–1km)	N(nodes)
Bangkok, Thailand	7.3	2.5	0.3	0.3	150k
Cebu City, Philippines	6.6	2.6	0.3	0.3	12k
Raleigh, United States	5.6	2.6	0.2	0.2	51k
Caracas, Venezuela	5.3	2.8	0.2	0.3	12k
Palembang, Indonesia	5.2	2.6	0.3	0.2	39k
Kampala, Uganda	5.1	2.6	0.2	0.2	15k
San Salvador, El Salvador	5.0	2.7	0.2	0.2	17k
Medan, Indonesia	4.9	2.6	0.3	0.2	64k
Pune, India	4.9	2.7	0.2	0.2	31k
Rawang, Malaysia	4.7	2.9	0.1	0.3	5.1k
Ho Chi Minh City, Viet Nam	4.6	2.7	0.2	0.2	64k
Baku, Azerbaijan	4.6	2.7	0.2	0.2	23k
Lagos, Nigeria	4.5	2.7	0.2	0.2	65k
Ulaanbaatar, Mongolia	4.5	2.7	0.2	0.2	10k
Cirebon, Indonesia	4.5	2.7	0.2	0.2	11k
Guatemala City, Guatemala	4.5	2.9	0.2	0.2	34k
Tijuana, Mexico	4.4	2.9	0.2	0.2	33k
Manila, Philippines	4.4	2.9	0.1	0.3	120k
Belgrade, Serbia	4.2	2.7	0.2	0.2	15k
Algiers, Algeria	4.1	2.9	0.2	0.2	31k
Florianopolis, Brazil	4.1	2.8	0.2	0.2	13k
Dhaka, Bangladesh	4.0	2.8	0.2	0.2	26k
Manchester, United Kingdom	4.0	2.7	0.2	0.2	74k
Ibadan, Nigeria	4.0	2.7	0.2	0.2	47k
Kolkata, India	3.9	2.8	0.2	0.2	36k
Coimbatore, India	3.9	2.8	0.2	0.2	16k
Los Angeles, United States	3.9	2.9	0.1	0.2	290k
Houston, United States	3.9	2.9	0.1	0.2	160k
Accra, Ghana	3.9	2.8	0.2	0.2	57k
Gainesville, United States	3.8	3.0	0.2	0.2	5.8k
Cleveland, United States	3.8	2.9	0.1	0.2	47k
Johannesburg, South Africa	3.8	2.9	0.1	0.2	110k
Ipoh, Malaysia	3.8	2.9	0.1	0.2	16k
Springfield, United States	3.7	2.9	0.2	0.2	19k
Mumbai, India	3.7	2.8	0.2	0.2	34k
Kabul, Afghanistan	3.6	2.8	0.2	0.2	21k
Shymkent, Kazakhstan	3.5	2.9	0.2	0.2	9.5k
Sheffield, United Kingdom	3.5	2.7	0.2	0.2	32k
London, United Kingdom	3.4	2.8	0.1	0.2	180k
Portland, United States	3.4	2.9	0.1	0.2	67k

	Sprawl (SNDi)	Nodal degree	Frc non-cycle (length)	Log Circuity (0.5–1km)	N(nodes)
Modesto, United States	3.4	2.8	0.1	0.2	13k
Killeen, United States	3.4	2.9	0.1	0.2	8k
Quito, Ecuador	3.3	2.9	0.1	0.2	32k
Auckland, New Zealand	3.3	2.9	0.1	0.2	20k
Sydney, Australia	3.3	2.9	0.1	0.2	76k
Palmas, Brazil	3.3	3.0	0.1	0.2	5.3k
Minneapolis, United States	3.3	3.0	0.1	0.2	88k
Victoria, Canada	3.3	2.9	0.1	0.2	8.8k
Tashkent, Uzbekistan	3.3	2.9	0.1	0.2	30k
Tangshan, China	3.3	2.9	0.1	0.2	5.6k
Vijayawada, India	3.2	2.9	0.2	0.2	9k
New York, United States	3.1	2.9	0.1	0.2	360k
Jaipur, India	3.1	2.9	0.1	0.2	40k
Hyderabad, India	3.1	2.9	0.1	0.2	140k
Chicago, United States	2.9	3.0	0.1	0.2	210k
Milan, Italy	2.9	2.9	0.1	0.2	140k
Toledo, United States	2.8	3.0	0.1	0.2	16k
Philadelphia, United States	2.8	3.0	0.1	0.2	160k
Guangzhou, China	2.8	3.0	0.1	0.2	73k
Port Elizabeth, South Africa	2.7	3.0	0.1	0.2	16k
Mexico City, Mexico	2.7	3.0	0.1	0.2	190k
Kinshasa, DR Congo	2.7	3.1	0.1	0.2	46k
Shenzhen, China	2.7	3.1	0.1	0.2	23k
Ahmedabad, India	2.7	2.9	0.1	0.2	12k
Tehran, Iran	2.6	2.9	0.1	0.2	82k
Palermo, Italy	2.6	2.9	0.2	0.1	11k
Cabimas, Venezuela	2.6	2.9	0.1	0.2	8.4k
Astrakhan, Russia	2.5	3.0	0.1	0.2	6.2k
Yamaguchi, Japan	2.5	2.9	0.1	0.2	12k
Chengdu, China	2.5	3.1	0.1	0.2	21k
Lahore, Pakistan	2.5	3.0	0.1	0.2	40k
Guadalajara, Mexico	2.5	3.1	0.1	0.2	75k
Sao Paulo, Brazil	2.5	3.0	0.1	0.2	180k
Curitiba, Brazil	2.4	3.1	0.1	0.2	45k
Ahvaz, Iran	2.4	3.1	0.1	0.2	13k
Zhengzhou, China	2.4	3.2	0.1	0.2	9.4k
Qingdao, China	2.4	3.0	0.1	0.2	19k
Addis Ababa, Ethiopia	2.3	3.0	0.1	0.2	43k
Beijing, China	2.3	3.1	0.1	0.2	39k
Tianjin, China	2.3	3.1	0.1	0.2	20k
Wuhan, China	2.3	3.1	0.1	0.2	15k
Moscow, Russia	2.2	3.2	0.1	0.2	54k

	Sprawl (SNDi)	Nodal degree	Frc non-cycle (length)	Log Circuity (0.5–1km)	N(nodes)
Qom, Iran	2.2	3.0	0.1	0.2	11k
Reynosa, Mexico	2.2	3.2	0.1	0.2	11k
Baghdad, Iraq	2.2	3.1	0.1	0.2	47k
Warsaw, Poland	2.2	3.1	0.1	0.2	29k
Kanpur, India	2.1	3.0	0.1	0.2	5.5k
Cairo, Egypt	2.1	3.1	0.1	0.2	62k
Riyadh, Saudi Arabia	2.1	3.1	0.0	0.2	90k
Shanghai, China	2.1	3.2	0.1	0.2	36k
Hangzhou, China	2.0	3.2	0.1	0.2	22k
Tel Aviv, Israel	2.0	3.1	0.1	0.1	28k
Gombe, Nigeria	2.0	3.1	0.1	0.2	6.9k
Changzhou, China	2.0	3.2	0.1	0.2	5.7k
Paris, France	2.0	3.1	0.1	0.1	140k
Montreal, Canada	1.9	3.2	0.1	0.2	52k
Santiago, Chile	1.9	3.1	0.1	0.1	93k
Antwerp, Belgium	1.9	3.1	0.1	0.1	23k
Singapore, Singapore	1.9	3.3	0.1	0.2	17k
Saint Petersburg, Russia	1.9	3.3	0.1	0.2	20k
Karachi, Pakistan	1.8	3.2	0.1	0.2	36k
Belo Horizonte, Brazil	1.8	3.2	0.1	0.2	50k
Luanda, Angola	1.8	3.1	0.1	0.1	51k
Jinan, China	1.7	3.2	0.1	0.2	5.9k
Kayseri, Turkey	1.7	3.1	0.1	0.2	12k
Okayama, Japan	1.6	3.1	0.1	0.1	56k
Malatya, Turkey	1.6	3.1	0.1	0.1	5.3k
Cochabamba, Bolivia	1.6	3.2	0.1	0.1	21k
Istanbul, Turkey	1.5	3.2	0.1	0.1	140k
Lubumbashi, DR Congo	1.5	3.1	0.1	0.1	17k
Culiacan, Mexico	1.5	3.3	0.1	0.1	15k
Sana, Yemen	1.5	3.1	0.1	0.1	11k
Busan, South Korea	1.4	3.2	0.1	0.1	24k
Ribeirao Preto, Brazil	1.4	3.3	0.1	0.1	12k
Bogota, Colombia	1.3	3.2	0.1	0.1	50k
Marrakesh, Morocco	1.3	3.2	0.1	0.1	12k
Osaka, Japan	1.3	3.2	0.1	0.1	180k
Seoul, South Korea	1.2	3.2	0.1	0.1	130k
Fukuoka, Japan	1.2	3.2	0.1	0.1	56k
Taipei, China	1.2	3.2	0.1	0.1	33k
Jinju, South Korea	1.2	3.2	0.1	0.1	5.1k
Budapest, Hungary	1.2	3.2	0.1	0.1	34k
Alexandria, Egypt	1.1	3.2	0.1	0.1	11k
Tokyo, Japan	1.1	3.1	0.1	0.1	800k

	Sprawl (SNDi)	Nodal degree	Frc non-cycle (length)	Log Circuity (0.5–1km)	N(nodes)
Bamako, Mali	1.1	3.2	0.1	0.1	36k
Madrid, Spain	1.1	3.3	0.1	0.1	55k
Cordoba, Argentina	1.0	3.3	0.0	0.1	29k
Vienna, Austria	1.0	3.3	0.1	0.1	23k
Berlin, Germany	0.9	3.3	0.0	0.1	40k
Hong Kong, China	0.9	3.4	0.0	0.1	6.1k
Gwangju, South Korea	0.9	3.3	0.1	0.1	10k
Cheonan, South Korea	0.8	3.3	0.1	0.1	5.3k
Thessaloniki, Greece	0.5	3.3	0.1	0.1	14k
Khartoum, Sudan	0.5	3.3	0.0	0.1	110k
Buenos Aires, Argentina	0.3	3.5	0.0	0.1	160k

M.3 Plots of cluster examples

The clusters exemplified (one page, or 12 examples, per cluster) below include two that are excluded from the main text, which are numbered 0 and 3.

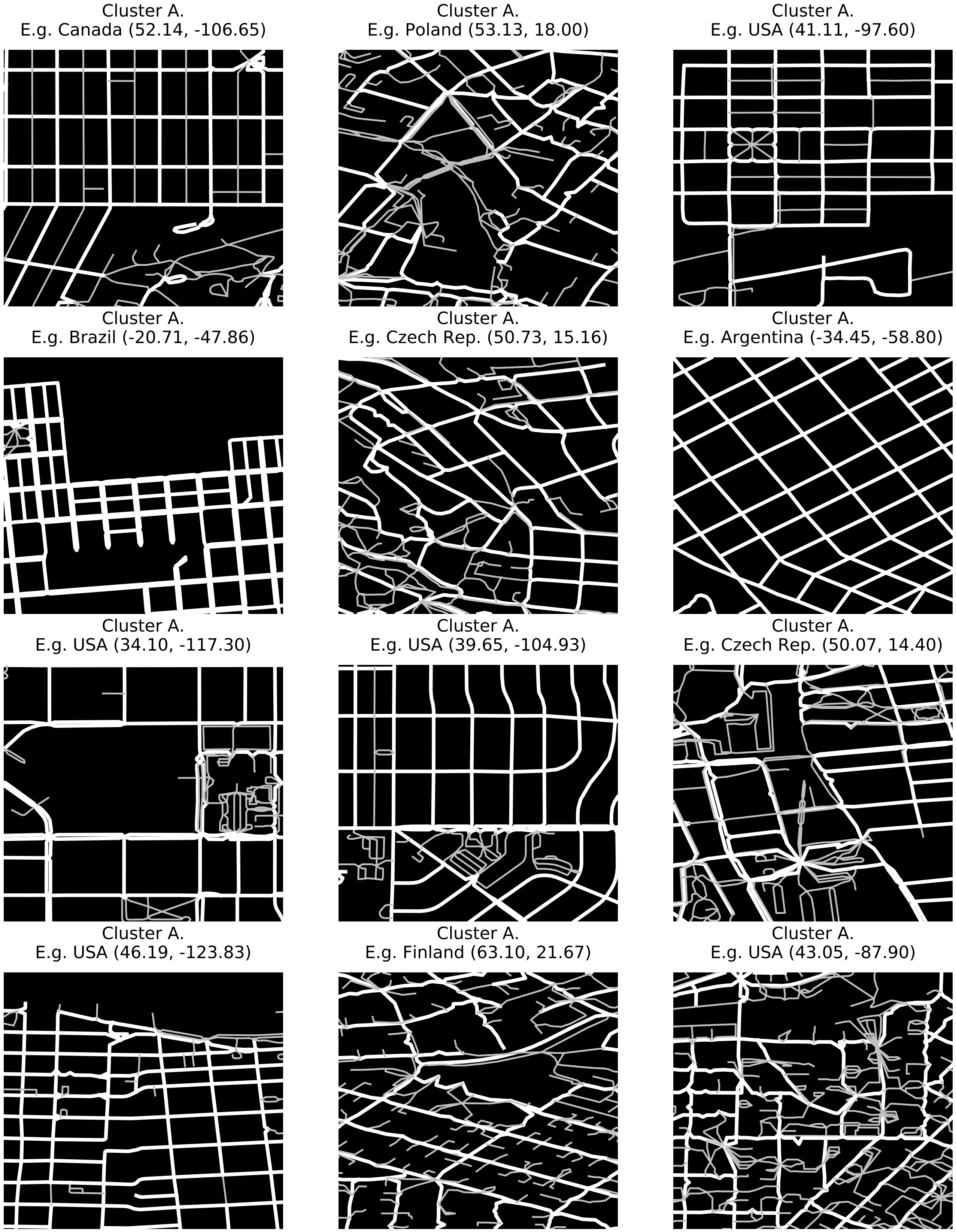
Cluster A.
E.g. Canada (52.14, -106.65)
Cluster A.
E.g. Poland (53.13, 18.00)
Cluster A.
E.g. USA (41.11, -97.60)
Cluster A.
E.g. Brazil (-20.71, -47.86)
Cluster A.
E.g. Czech Rep. (50.73, 15.16)
Cluster A.
E.g. Argentina (-34.45, -58.80)
Cluster A.
E.g. USA (34.10, -117.30)
Cluster A.
E.g. USA (39.65, -104.93)
Cluster A.
E.g. Czech Rep. (50.07, 14.40)
Cluster A.
E.g. USA (46.19, -123.83)
Cluster A.
E.g. Finland (63.10, 21.67)
Cluster A.
E.g. USA (43.05, -87.90)

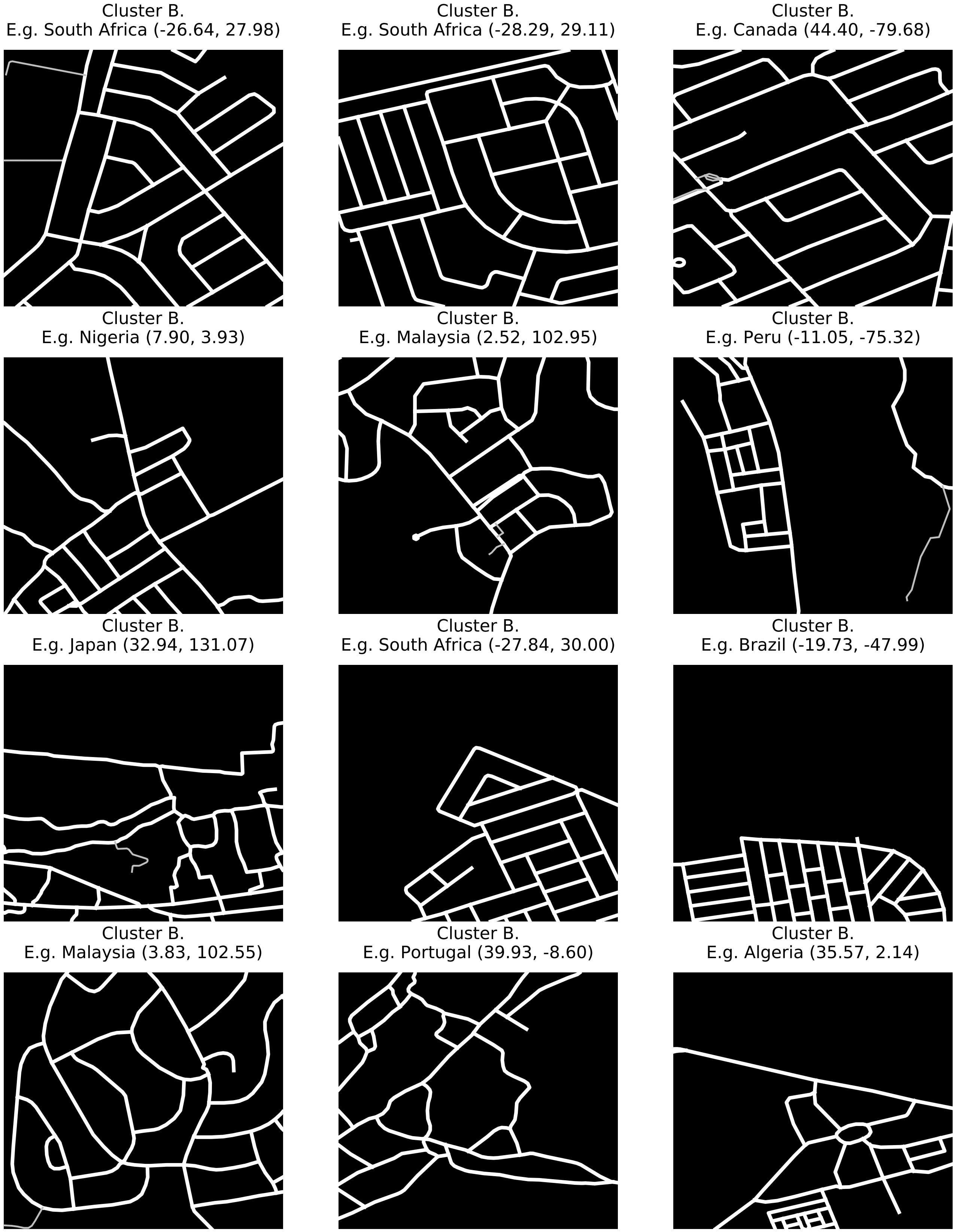
Cluster B.
E.g. South Africa (-26.64, 27.98)
Cluster B.
E.g. South Africa (-28.29, 29.11)
Cluster B.
E.g. Canada (44.40, -79.68)
Cluster B.
E.g. Nigeria (7.90, 3.93)
Cluster B.
E.g. Malaysia (2.52, 102.95)
Cluster B.
E.g. Peru (-11.05, -75.32)
Cluster B.
E.g. Japan (32.94, 131.07)
Cluster B.
E.g. South Africa (-27.84, 30.00)
Cluster B.
E.g. Brazil (-19.73, -47.99)
Cluster B.
E.g. Malaysia (3.83, 102.55)
Cluster B.
E.g. Portugal (39.93, -8.60)
Cluster B.
E.g. Algeria (35.57, 2.14)

Cluster C.
E.g. France (45.15, 5.74)

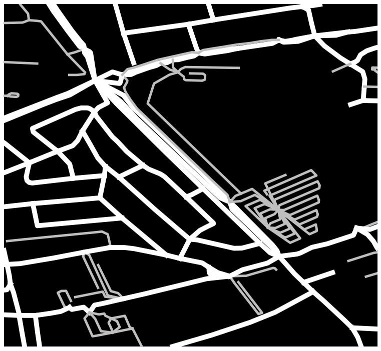

Cluster C.
E.g. USA (40.64, -103.20)

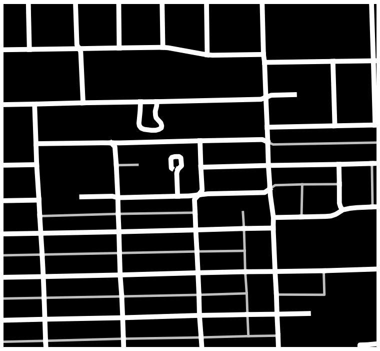

Cluster C.
E.g. Netherlands (52.30, 6.82)

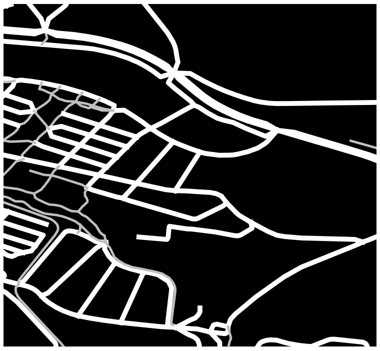

Cluster C.
E.g. Australia (-34.85, 138.53)

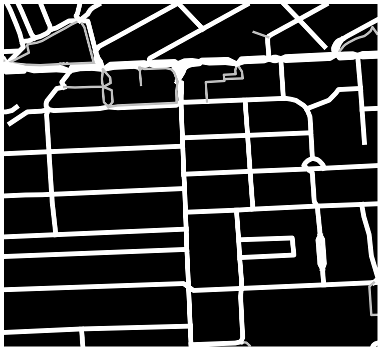

Cluster C.
E.g. Chile (-33.60, -70.58)

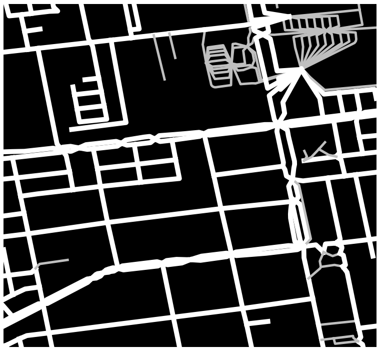

Cluster C.
E.g. Poland (52.15, 20.71)

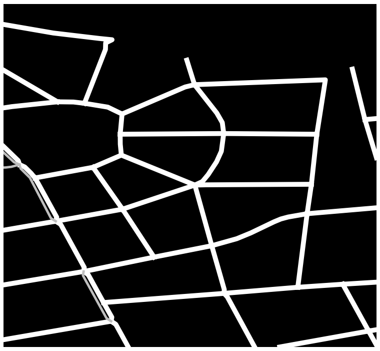

Cluster C.
E.g. Algeria (35.60, 1.81)

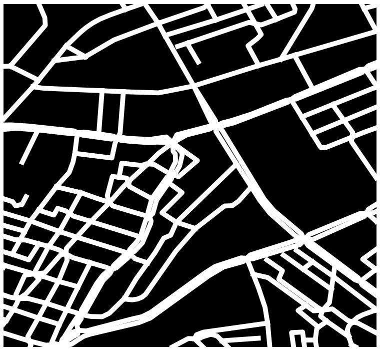

Cluster C.
E.g. Taiwan (24.17, 120.70)

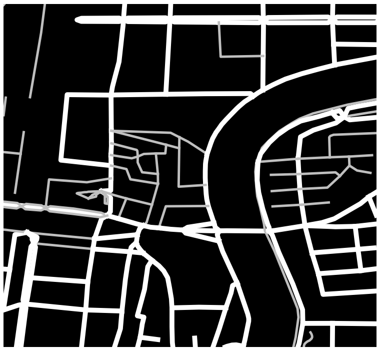

Cluster C.
E.g. Canada (46.55, -75.50)

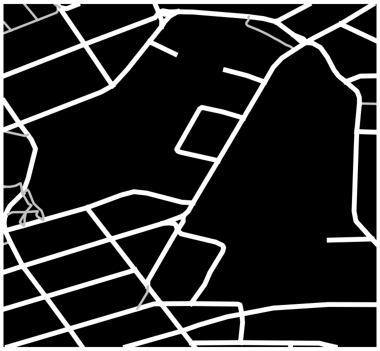

Cluster C.
E.g. China (23.01, 113.39)

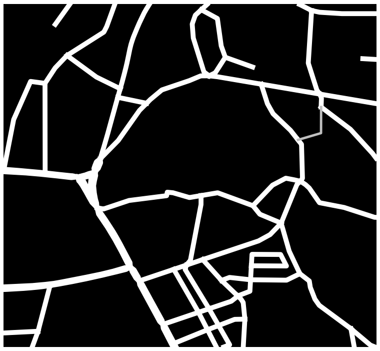

Cluster C.
E.g. India (12.32, 76.65)

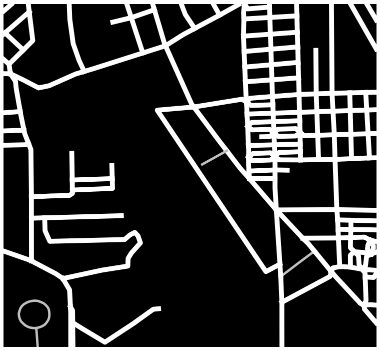

Cluster C.
E.g. Japan (34.79, 135.69)

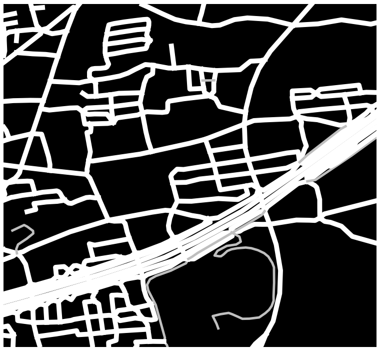

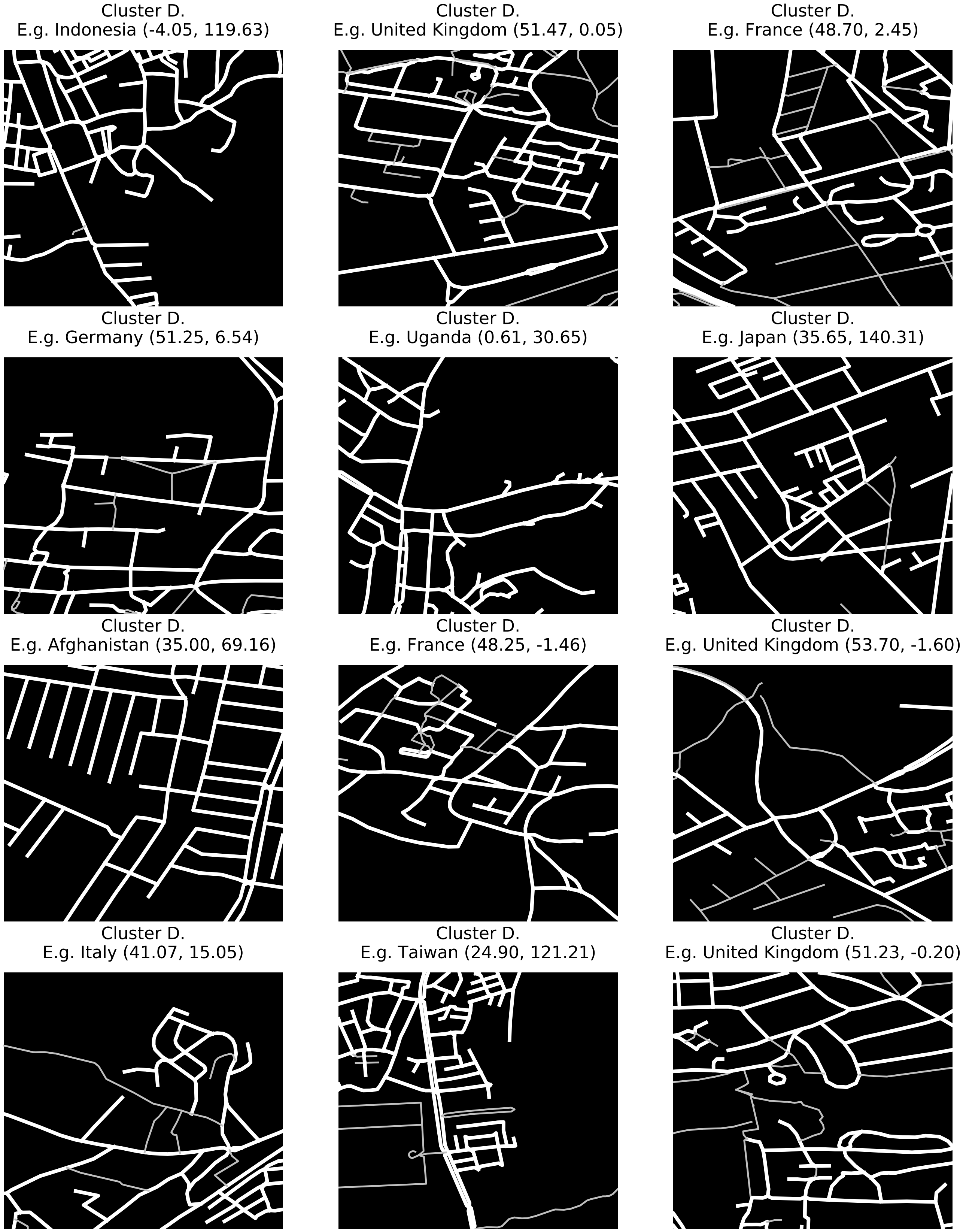
Cluster D.
E.g. Indonesia (-4.05, 119.63)
Cluster D.
E.g. United Kingdom (51.47, 0.05)
Cluster D.
E.g. France (48.70, 2.45)
Cluster D.
E.g. Germany (51.25, 6.54)
Cluster D.
E.g. Uganda (0.61, 30.65)
Cluster D.
E.g. Japan (35.65, 140.31)
Cluster D.
E.g. Afghanistan (35.00, 69.16)
Cluster D.
E.g. France (48.25, -1.46)
Cluster D.
E.g. United Kingdom (53.70, -1.60)
Cluster D.
E.g. Italy (41.07, 15.05)
Cluster D.
E.g. Taiwan (24.90, 121.21)
Cluster D.
E.g. United Kingdom (51.23, -0.20)

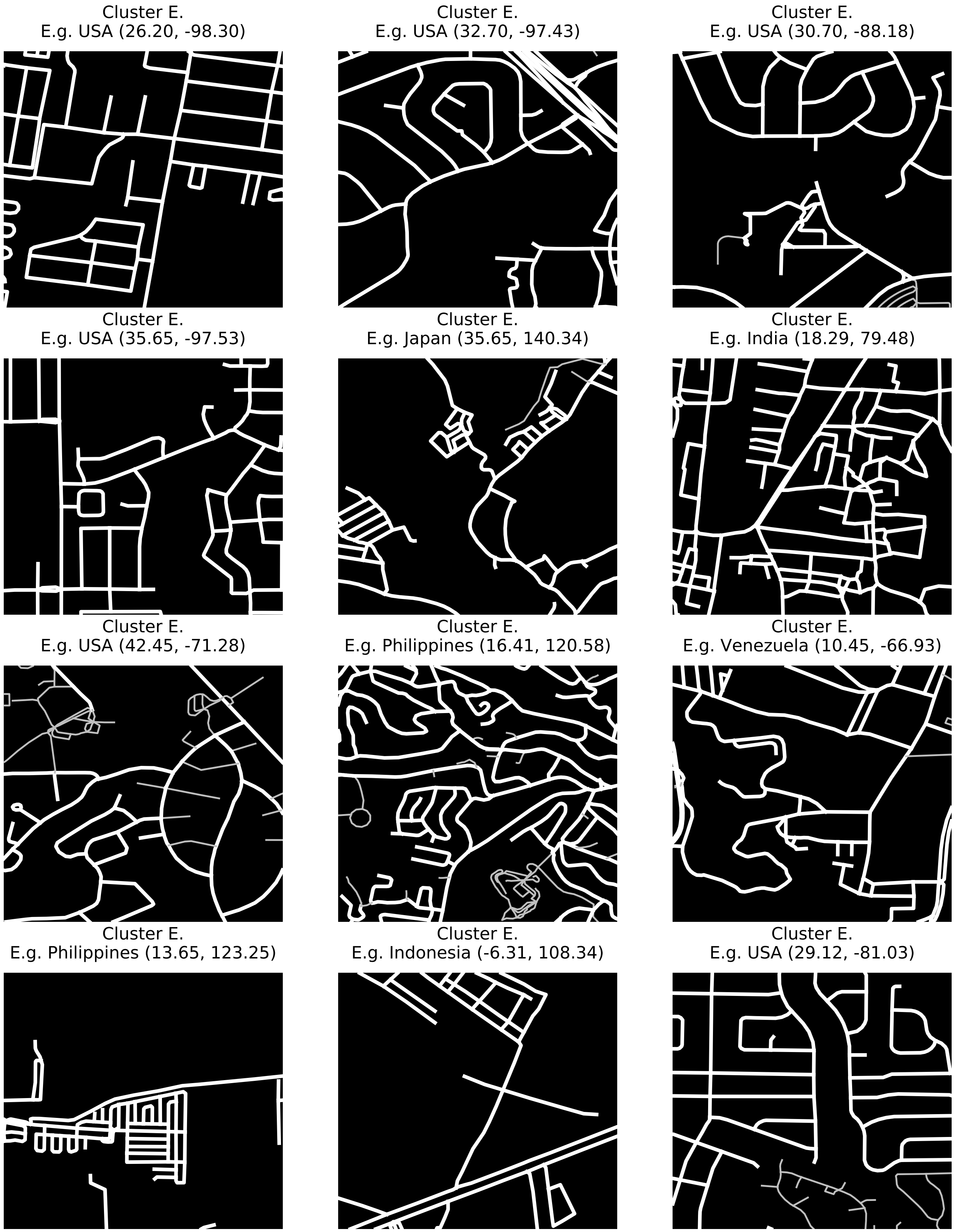
Cluster E.
E.g. USA (26.20, -98.30)
Cluster E.
E.g. USA (32.70, -97.43)
Cluster E.
E.g. USA (30.70, -88.18)
Cluster E.
E.g. USA (35.65, -97.53)
Cluster E.
E.g. Japan (35.65, 140.34)
Cluster E.
E.g. India (18.29, 79.48)
Cluster E.
E.g. USA (42.45, -71.28)
Cluster E.
E.g. Philippines (16.41, 120.58)
Cluster E.
E.g. Venezuela (10.45, -66.93)
Cluster E.
E.g. Philippines (13.65, 123.25)
Cluster E.
E.g. Indonesia (-6.31, 108.34)
Cluster E.
E.g. USA (29.12, -81.03)

Cluster F.
E.g. United Kingdom (52.06, 1.26)

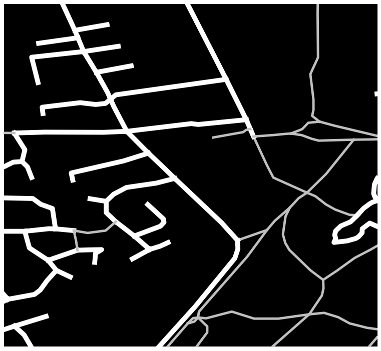

Cluster F.
E.g. India (26.20, 91.71)

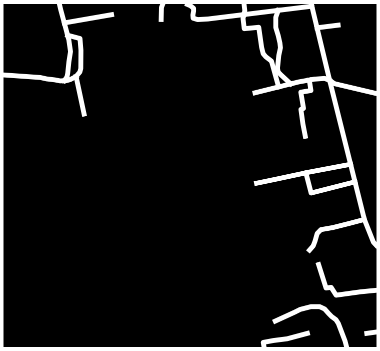

Cluster F.
E.g. United Kingdom (53.55, -0.14)

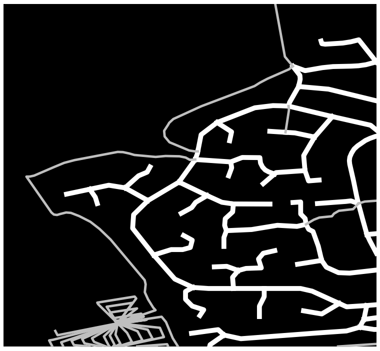

Cluster F.
E.g. France (45.55, 5.53)

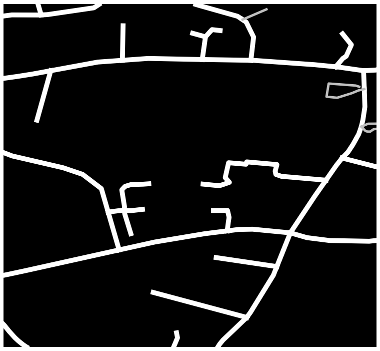

Cluster F.
E.g. Portugal (41.00, -8.60)

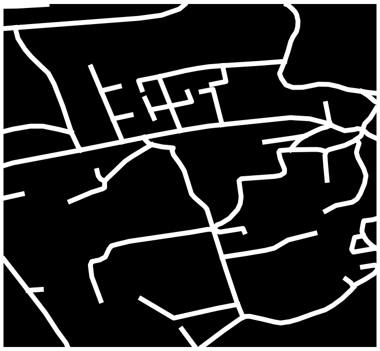

Cluster F.
E.g. United Kingdom (52.39, -2.01)

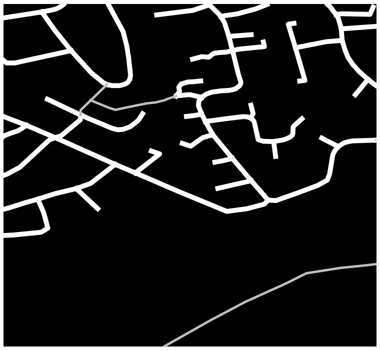

Cluster F.
E.g. Uganda (0.60, 30.64)

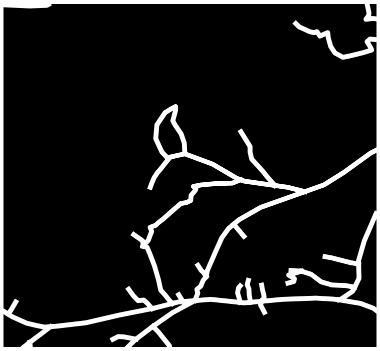

Cluster F.
E.g. Indonesia (-5.34, 119.50)

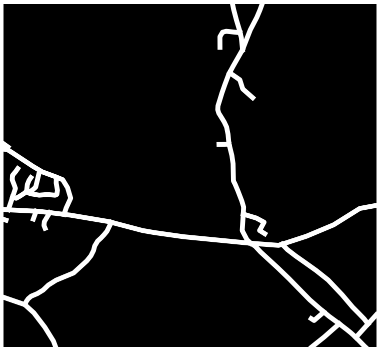

Cluster F.
E.g. Indonesia (-2.69, 111.65)

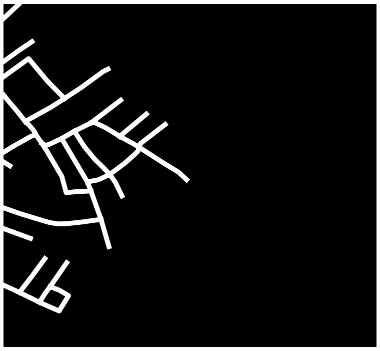

Cluster F.
E.g. Viet Nam (11.01, 106.42)

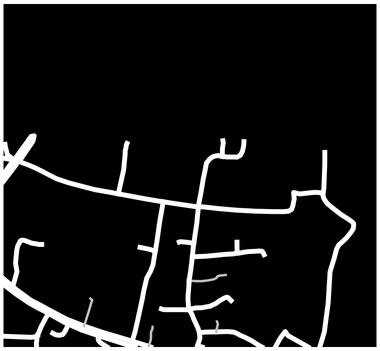

Cluster F.
E.g. Viet Nam (15.12, 108.82)

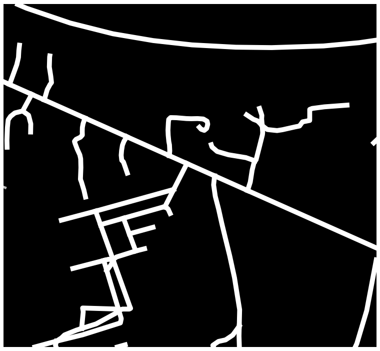

Cluster F.
E.g. Sri Lanka (5.94, 80.51)

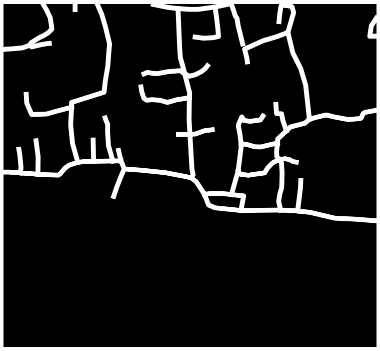

Cluster 6.
E.g. China (37.42, 116.35)

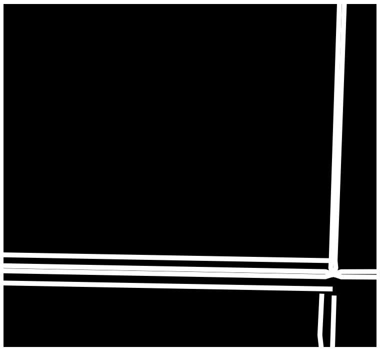

Cluster 6.
E.g. USA (39.97, -79.81)

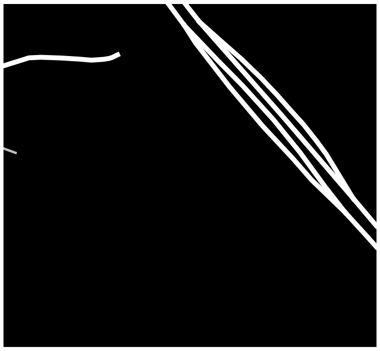

Cluster 6.
E.g. Nigeria (12.34, 13.55)

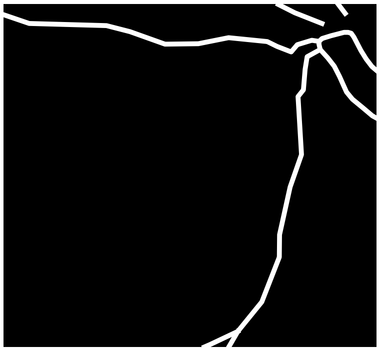

Cluster 6.
E.g. India (18.13, 74.30)

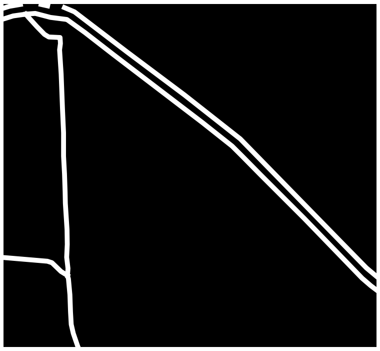

Cluster 6.
E.g. China (34.80, 112.48)

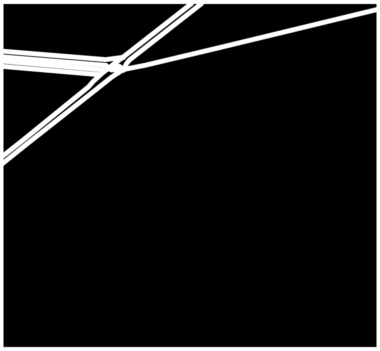

Cluster 6.
E.g. China (35.02, 104.70)

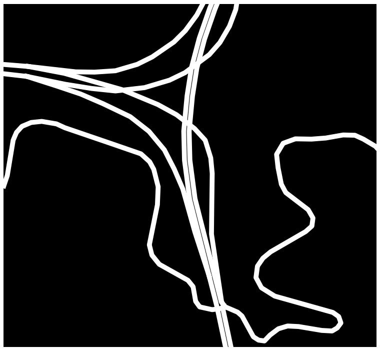

Cluster 6.
E.g. India (9.50, 78.11)

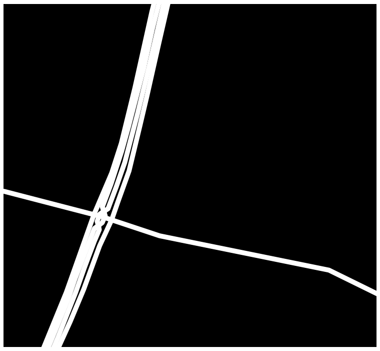

Cluster 6.
E.g. China (30.40, 103.97)

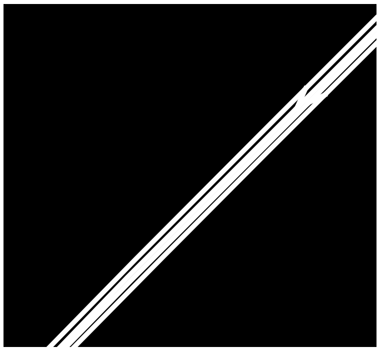

Cluster 6.
E.g. USA (35.21, -106.58)

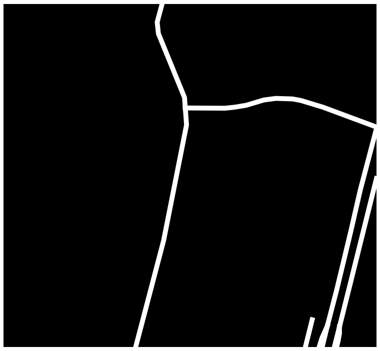

Cluster 6.
E.g. Egypt (30.49, 32.10)

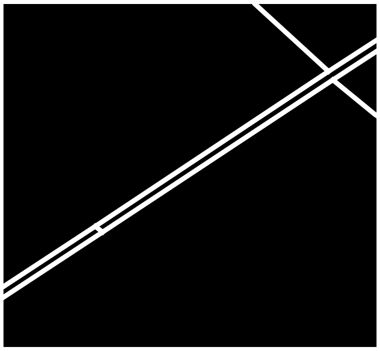

Cluster 6.
E.g. China (37.40, 121.68)

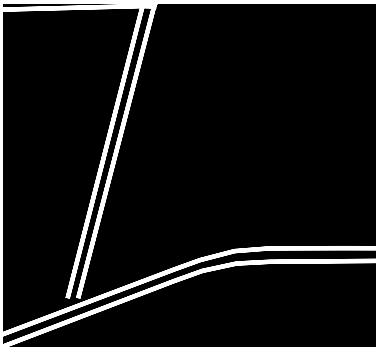

Cluster 6.
E.g. Saudi Arabia (18.26, 42.75)

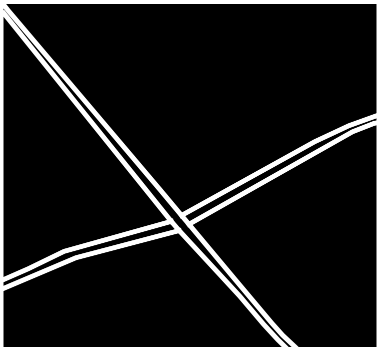

Cluster G.
E.g. Brazil (-27.00, -49.10)

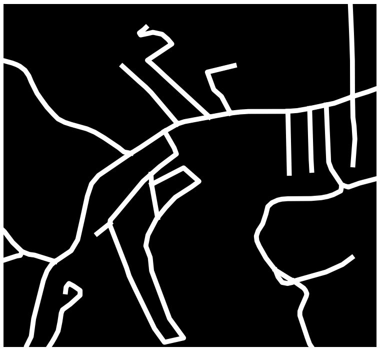

Cluster G.
E.g. USA (38.15, -76.51)

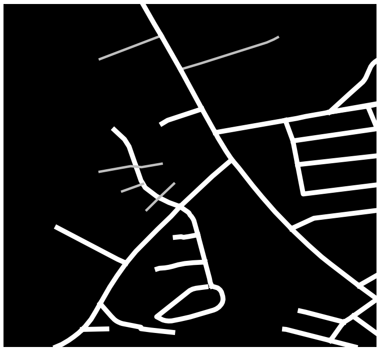

Cluster G.
E.g. USA (32.93, -109.20)

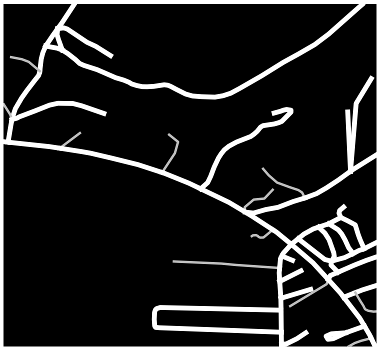

Cluster G.
E.g. USA (42.84, -70.85)

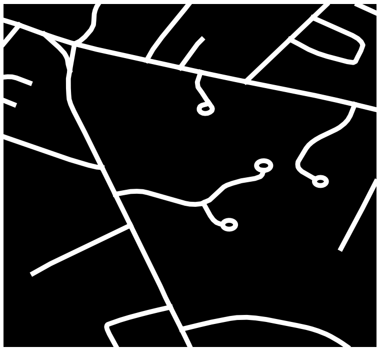

Cluster G.
E.g. USA (34.99, -85.10)

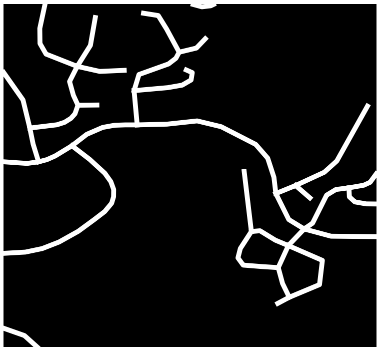

Cluster G.
E.g. Poland (52.41, 21.40)

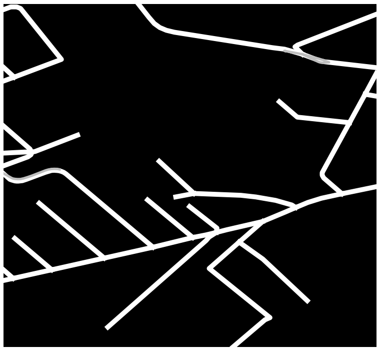

Cluster G.
E.g. Egypt (30.95, 32.47)

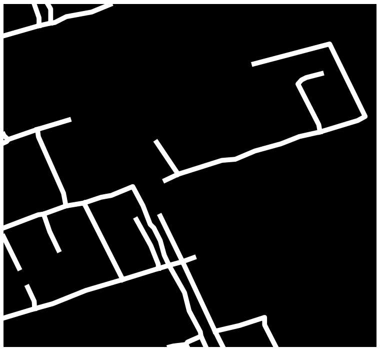

Cluster G.
E.g. USA (39.45, -80.17)

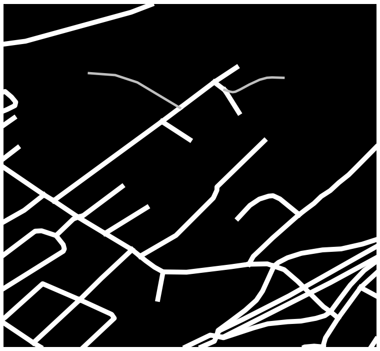

Cluster G.
E.g. USA (34.11, -81.25)

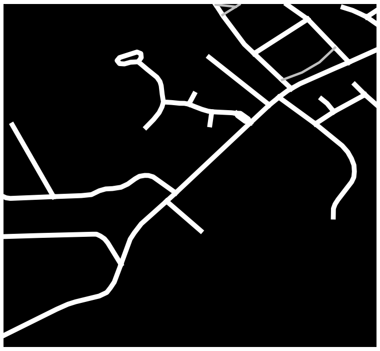

Cluster G.
E.g. South Africa (-29.43, 31.25)

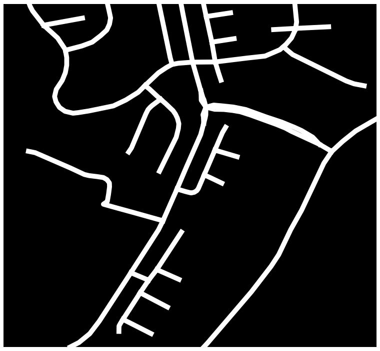

Cluster G.
E.g. Mozambique (-10.99, 40.48)

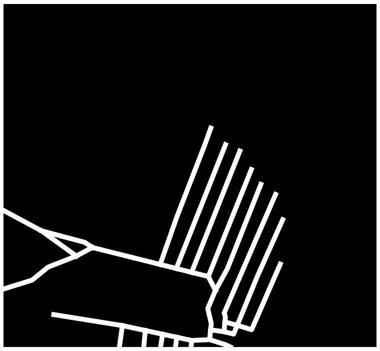

Cluster G.
E.g. USA (31.85, -110.96)

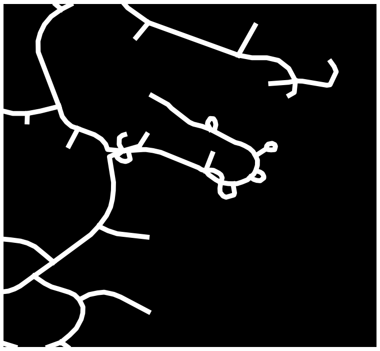

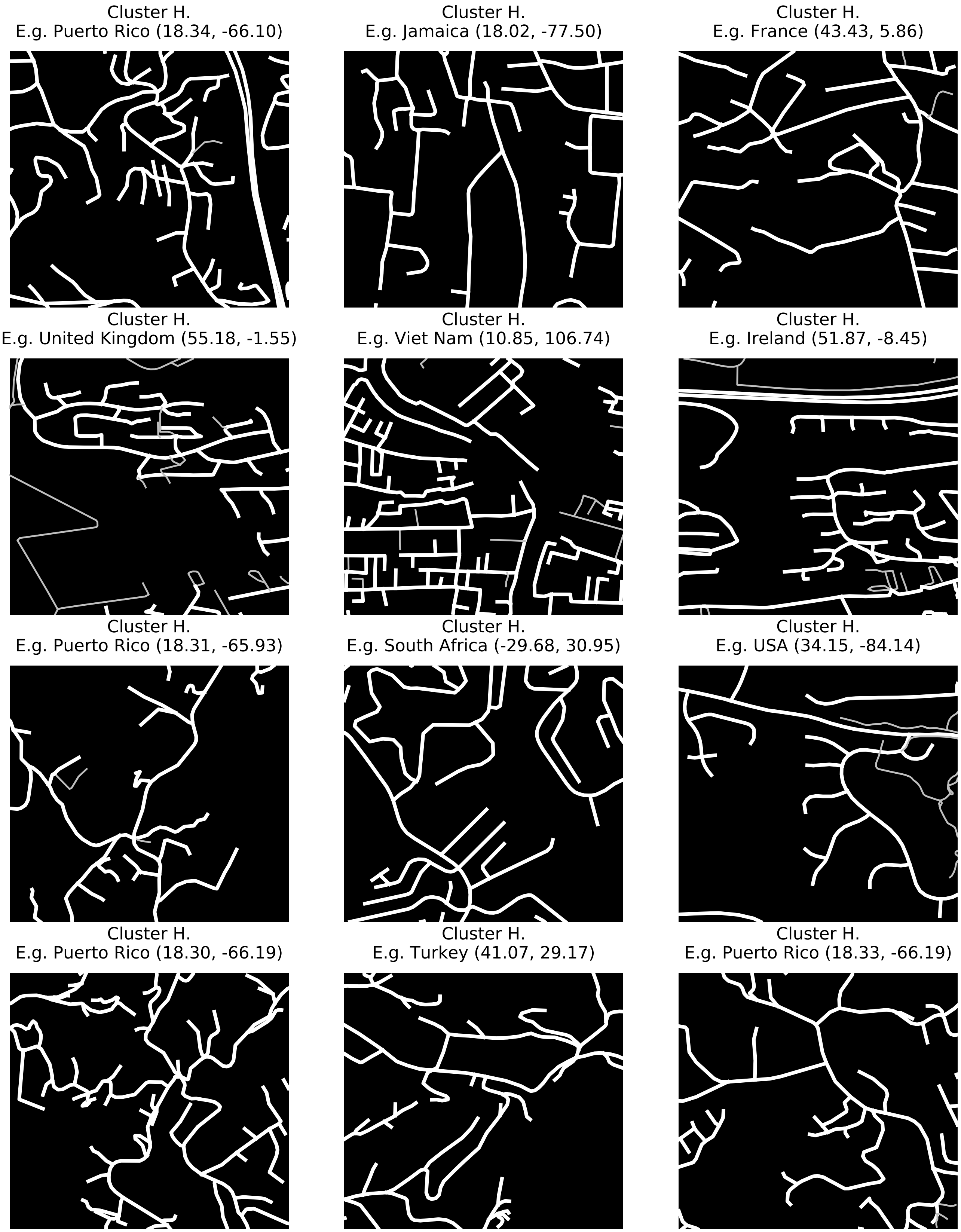

Cluster H.
E.g. Puerto Rico (18.34, -66.10)
Cluster H.
E.g. Jamaica (18.02, -77.50)
Cluster H.
E.g. France (43.43, 5.86)
Cluster H.
E.g. United Kingdom (55.18, -1.55)
Cluster H.
E.g. Viet Nam (10.85, 106.74)
Cluster H.
E.g. Ireland (51.87, -8.45)
Cluster H.
E.g. Puerto Rico (18.31, -65.93)
Cluster H.
E.g. South Africa (-29.68, 30.95)
Cluster H.
E.g. USA (34.15, -84.14)
Cluster H.
E.g. Puerto Rico (18.30, -66.19)
Cluster H.
E.g. Turkey (41.07, 29.17)
Cluster H.
E.g. Puerto Rico (18.33, -66.19)

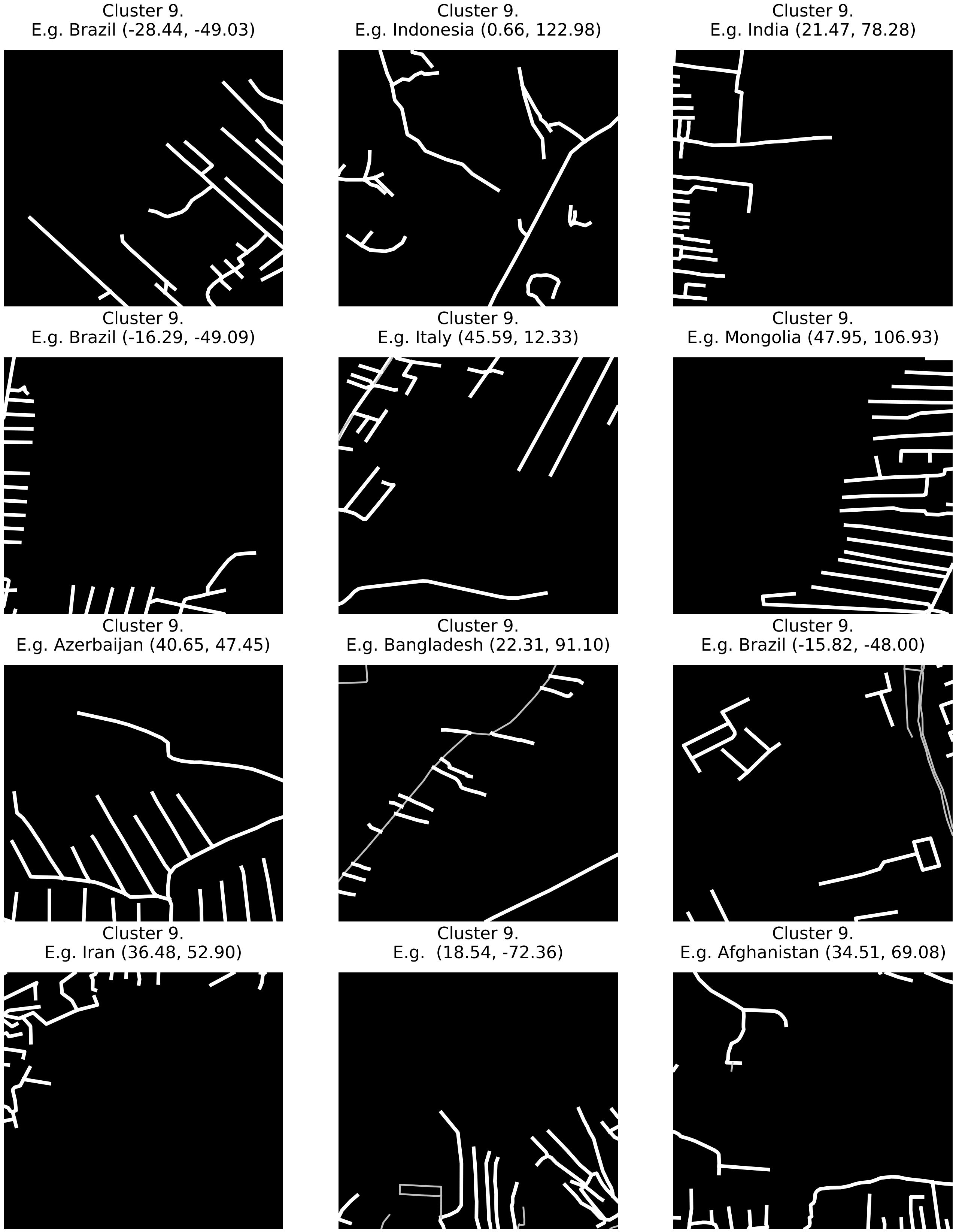
Cluster 9.
E.g. Brazil (-28.44, -49.03)
Cluster 9.
E.g. Indonesia (0.66, 122.98)
Cluster 9.
E.g. India (21.47, 78.28)
Cluster 9.
E.g. Brazil (-16.29, -49.09)
Cluster 9.
E.g. Italy (45.59, 12.33)
Cluster 9.
E.g. Mongolia (47.95, 106.93)
Cluster 9.
E.g. Azerbaijan (40.65, 47.45)
Cluster 9.
E.g. Bangladesh (22.31, 91.10)
Cluster 9.
E.g. Brazil (-15.82, -48.00)
Cluster 9.
E.g. Iran (36.48, 52.90)
Cluster 9.
E.g. (18.54, -72.36)
Cluster 9.
E.g. Afghanistan (34.51, 69.08)

M.4 World maps

World maps for our sprawl metrics are available at:
https://sprawlmap.org
