# Supplementary material for: A global assessment of street-network sprawl
Source: PLoS One. 2019 Nov 26;14(11):e0223078. doi: 10.1371/journal.pone.0223078 (PMC6879130; doi:10.1371/journal.pone.0223078)
Supplement: Attachment — Submitted filename: reviews-responses.pdf [file pone.0223078.s003.pdf]

Dear Dr. Kato,

Thank you for the opportunity to submit a revised version of our manuscript, "A global assessment of street-network sprawl," to PLOS ONE. We thank the reviewers for their comments, and believe that the revisions have strengthened the paper. We provide a point-by-point response below, and a version of the manuscript with changes tracked.

Regarding the editorial issues raised:

- Google Maps and Streetview copyright: Thank you for drawing the copyright issue to our attention, i.e. that the CC license used by PLOS does not allow for use of Google imagery. After discussions with the PLOS editorial staff, we have removed the Streetview and satellite imagery from Figure 3 and SI Table S4, as there are no suitable alternatives. Instead, we have retained the OpenStreetMap-based images, and provided hyperlinks from the figure to the satellite and Streetview imagery so that readers still have easy access. We have also hyperlinked to the composite figure on our website.

- We have included captions for our Supporting Information files at the end of the manuscript, and updated any in-text citations to match accordingly.

We also thank you for your invitation to be considered under the "Urban Ecosystems" Call for Papers, and we would be happy to be included in this collection should our manuscript be accepted. We understand from discussions with PLOS editorial staff that this will not have any impact on the timelines for review or acceptance decisions.

Thank you for your consideration.

Chris Barrington-Leigh and Adam Millard-Ball

Comments from Reviewer 2

Actually this paper shows original and interesting classification such as Figure 4, linking between real road type and road data from Open Street Map. This classification has very important meanings that can be easily evaluated by global data such as OSM. The statistical result looks feasible and realistic. So I like this paper and can accept without revision.

AUTHORS' RESPONSE: Thank you. We are glad that you like the paper!

Comments from Reviewer 1

This is an interesting paper and does a nice job building on the authors' past work to explore street networks. It requires some revision to be suitable for publication.

AUTHORS' RESPONSE: Thank you.

In section 2.2 you refer to "sinuosity" as an indicator, but in table 1 you refer to "curviness." Are these the same indicator? Clarification needed.

AUTHORS' RESPONSE: Yes, they are the same. We have updated the table accordingly.

I'm curious about the motivation to use only the first PCA component as the index, when it only explains about one-third of the variance. I can understand the desire for a single-dimensional indicator, rather than two or three, but what is being lost or ignored if it only explains 36% of the variance? How effective of an indicator is it in the end? I would like to see this more clearly unpacked and motivated as a standalone indicator.

AUTHORS' RESPONSE: We are interested in the first PCA component as, in contrast to the other components, it unambiguously indicates sprawl, through loading positively on all our metrics. Other components indicate different aspects of street network design and connectivity which are beyond the scope of this paper. However, we publish the results for other components, and so other interested researchers may wish to pursue this line of inquiry. We see PCA as a tool to increase the interpretability of many related variables, and in this case a single component best achieves that goal.

We have added text as follows (further discussion is in the SI): "The second component, PCA_2, in contrast, emphasizes differences between our circuity measures and our other measures of connectivity. Rather than pointing unambiguously in the direction of increasing sprawl, high PCA_2 can be interpreted as a place where circuity is much greater than would be expected from nodal degree, dendricity, and sinuosity alone, such as a place where connected neighborhoods are separated by a river or steep topography."

See also our response below to the reviewer's comment about capturing a range of elements of street character.

The paper doesn't have a traditional literature review section per se, but references to the literature appear in some of the background developed in sections 1 and 2. It would be useful to see more clearly how this work is positioned in the research stream, including your own work. For example, this appears

to build on the past work of your own that you cite here in PNAS, ERL, and PLOS. Looking through those papers, I see some methodological and conceptual commonalities with this work, but also a nice trajectory leading to this project. It might be useful to explain how this all fits together.

This paper also has similarities to a couple of other street network analysis studies I saw recently, which should appear in your intro/methods sections, especially considering your call for larger sample sizes or global comparative work, which overlaps with theirs. The first did a descriptive analysis of American street networks and included some visuals similar to your figure 2: https://doi.org/10.1177/2399808318784595 The second was an analysis across countries/regions and included a clustering component to identify network design paradigms, similar to yours: https://doi.org/10.31235/osf.io/qj3p5 Your study does a nice job taking things a step further (with the auto-ownership analysis) than the descriptive/clustering analyses of those two papers, creating a nice next step in the overall literature trajectory.

On page 5 you mention empirical implementations of circuity measures (etc) being rare in the research literature and provide a couple exceptions. In addition, see also the two studies linked above (they also appear to operationalize a circuity indicator), as well as these other studies: https://doi.org/10.1068%2Fb130131p and https://doi.org/10.1016/S0965-8564(01)00044-1

AUTHORS' RESPONSE: Thank you for the suggestions and the links to additional references. We have made some additions as follows:

- We now note the progression from our earlier work in PNAS and ERL explicitly in the introduction.
- We have added the references to Boeing [2018] in both the main text and SI, including (in the SI) his point about the scale-dependence of circuity measures, and sourced the inspiration for our visuals to Jacobs [1995]. The other suggested Boeing reference appears to be a working paper, with the clustering focused on identifying types of *cities* rather than our small-scale (1km^2) street typology, and so we have not made this addition.
- We already credited the circuity measure of Giacomin and Levinson [2015] in the SI, but we have added this reference to the main text as well. The circuity measure of Ballou et al. [2002] is about circuity between travel between major cities, and has more limited relevance to our discussion of street-network sprawl; we have chosen to leave this out.

Regarding the reviewer's comment about the lack of a traditional literature review section, we understand from the PLOS ONE author guidelines that articles should have a brief review of the key literature in the introduction, rather than a stand-alone section. Therefore, we discuss the literature briefly in sections 1 and 2. We also provide additional detail from the literature in the SI, noting where our component metrics of street-network sprawl have been developed by other authors in the past. If the editor wishes, we would be happy to extend the discussion of key literature further or bring in some of the detail from the SI to the main text.

I like your multiple-scales circuity measure. Is that a new contribution in this paper? If so, it should be mentioned as such because it seems like a useful way of considering it.

AUTHORS' RESPONSE:

Thank you. We have several minor refinements to individual measures of street-network sprawl, such as quantifying circuity at multiple scales, and also using network bridges to capture dendricity. However, conceptually, these suggestions have already been made in the literature. We see our main contributions as (i) computational, in that we report street-network sprawl for the entire planet, and (ii) validated measurement, through introducing a single-dimensional measure of street-network sprawl and validating it in multiple geographic contexts. These are highlighted in the introduction.

Page 5: by "network bridge" do you mean a bridge in the graph theory sense? It might be worth making that explicitly, as "bridge" terminology can get overloaded when considering road networks (which have overpasses and underpasses).

AUTHORS' RESPONSE: Yes, we mean in the graph theoretic sense. We have clarified as follows: "a network bridge (i.e., a link between two otherwise-disconnected network components)".

Page 7: you mention merging compound intersections and other preprocessing steps that are not explained in the paper text, but presumably would substantially impact results. You don't need to reproduce everything from the SI here, but it would be helpful if you could explain how/why you did the key preprocessing steps for comprehensibility's sake. For example, how did you define and merge compound intersections? What parameters did you use? What do you mean by "annealing joints"? I think a short summary would do.

AUTHORS' RESPONSE: We have revised this sentence to both be clearer about what we are doing, in non-technical terms, and to make the motivation self-explanatory. We believe this allows us to leave the detailed methods to the SI.

Page 7: if your results examine urban areas only, what is the purpose of conducting all the data collection and analysis for the entire planet?

AUTHORS' RESPONSE: While we are interested in urban areas in the context of the present paper, other researchers may find the non-urban results of interest, and/or prefer to use a different definition of "urban."

Section 3.1.2: the qualitative assessment suggests that there's a lot more to street character beyond the network indicators you've quantified here. How does that affect the interpretation of your findings? What is being lost when we quantify "the urban" that simply does not always lend itself to quantitative measurement?

AUTHORS' RESPONSE:

We motivate our approach as a tool to compare disparate urban form in a scalable way, in order to compare development worldwide, both in cross-section and in changes. In order to be scalable, this approach is necessarily quantitative. Quantifiable measurements are also necessary for specifying transparent policy rules to address urban sprawl.

At the same time, we agree with the reviewer that many other important characteristics of urban design are left out of a scalar, quantitative index. Our manuscript addresses this in particular through its sections on "Qualitative appraisal" and "Empirical street-network types", and we have now added a paragraph to Section 3.1.2 as follows:

"Our qualitative assessment here and in the SI indicates that SNDi correlates with different aspects of walkability and street connectivity in a range of economic and geographic contexts. Numerous related aspects of street character and urban design highlighted by this assessment, such as sidewalk widths and street-facing doors and windows, lend themselves to more focused analysis in future work, but themselves are likely to be constrained by the overall connectivity of the network."

It's not clear in the SI why you chose to parameterize the cluster analysis with k=8. How was this selected and why? Is this preferable to something like hierarchical clustering that allows flexible cluster sizes and a more interpretative cut-off process for the cluster number? This needs to be more clearly motivated.

AUTHORS' RESPONSE: As recommended in the literature, we see cluster analysis as a descriptive and exploratory tool. We follow the advice in Jain [2010]: "Typically, k-means is run independently for different values of K and the partition that appears the most meaningful to the domain expert is selected." We ran clusters with $k \in \{8, 9, 10\}$ and chose the result that was most interpretable.

We use k-means clustering for three main reasons:

- Computational: Even our large server was unable to hierarchically/agglomeratively cluster the 3.7 million grid cells using popular algorithms in Python (e.g. sklearn.cluster.AgglomerativeClustering).
- Empirical track record: While there are many different clustering algorithms, no one algorithm is clearly superior, and Jain [2010] suggests that the popularity of k-means is due to ease of implementation, simplicity, efficiency, and empirical success.
- In contrast to a hierarchical clustering algorithm, a partitional algorithm such as k-means does not impose a hierarchical structure. There is no reason why one would expect an empirical typology of street networks to form a hierarchy, and k-means rules this neither in nor out.

We have added text to the SI to clarify these points as follows:

"Cluster analysis is a descriptive and exploratory tool. As recommended by Jain [2010], we ran the analysis with several values of k and chose the result that was most interpretable. We use k-means rather than other clustering algorithms for computational reasons, and because of its ease of implementation, simplicity, efficiency, and empirical success [Jain, 2010]. Moreover, in contrast to a hierarchical clustering algorithm, a partitional algorithm such as k-means does not impose a hierarchical structure."

Table 2: are your region fixed effects referring to the country? Or to subregion entities within the countries?

AUTHORS' RESPONSE: They refer to country, not subregion entities, and we have changed the terminology.

References

Ronald H Ballou, Handoko Rahardja, and Noriaki Sakai. Selected country circuity factors for road travel distance estimation. *Transportation Research Part A: Policy and Practice*, 36(9):843 – 848, 2002. ISSN 0965-8564. doi: https://doi.org/10.1016/S0965-8564(01)00044-1. URL http://www.sciencedirect.com/science/article/pii/S0965856401000441.

Geoff Boeing. A multi-scale analysis of 27,000 urban street networks: Every US city, town, urbanized area, and Zillow neighborhood. *Environment and Planning B: Urban Analytics and City Science*, Online First, 2018. ISSN 2399-8083, 2399-8091. doi: 10.1177/2399808318784595. URL http://journals.sagepub.com/doi/10.1177/2399808318784595.

David J. Giacomin and David M. Levinson. Road network circuity in metropolitan areas. *Environment & Planning B: Planning & Design*, 42:1040–1053, 2015. doi: 10.1068/b130131p.

Allan B. Jacobs. *Great streets.* MIT Press, Cambridge, Mass., new ed edition, 1995. ISBN 978-0-262-60023-1.

Anil K. Jain. Data clustering: 50 years beyond k-means. *Pattern Recognition Letters*, 31(8):651 – 666, 2010. ISSN 0167-8655. doi: https://doi.org/10.1016/j.patrec.2009.09.011. URL http://www.sciencedirect.com/science/article/pii/S0167865509002323. Award winning papers from the 19th International Conference on Pattern Recognition (ICPR).
