# Supplementary material for: A global assessment of street-network sprawl
Source: PLoS One. 2019 Nov 26;14(11):e0223078. doi: 10.1371/journal.pone.0223078 (PMC6879130; doi:10.1371/journal.pone.0223078)
Supplement: Attachment — Submitted filename: reviews-responses-round3.pdf [file pone.0223078.s004.pdf]

Dear Dr. Kato,

Thank you for the opportunity to submit a revised version of our manuscript, "A global assessment of street-network sprawl," to PLOS ONE. We thank Reviewer #1 for their comments, and respond as follows:

Reviewer comment: Reviewer: The revised manuscript looks good in general.

AUTHORS' RESPONSE: Thank you.

Reviewer comment: The author's response regarding the first PCA component makes sense. I'm still a little concerned about its low percent of variance explained (in terms of real-world interpretation), but at the same time I believe that your next text in the revision sufficiently caveats and explains your finding.

AUTHORS' RESPONSE: Thank you. We are glad that the additional text provides sufficient justification and caveats.

Reviewer comment: Regarding your comments on the lit review: it seems that this is the standard PLOS format and is therefore fine. However, I do believe that leaving out the reference to Ballou et al 2002 remains an oversight, especially considering your claim about the rarity of empirical implementations of circuity. I encourage you to reconsider.

AUTHORS' RESPONSE: We have added the Ballou et al 2002 reference to the main text.

Thank you for your consideration.

Chris Barrington-Leigh and Adam Millard-Ball
